# Optimized GM(0,N) model with exponential–trigonometric transformations and PSO for queue length prediction at metered roundabouts

**DOI:** 10.1038/s41598-026-48464-9

**Published:** 2026-05-15

**Authors:** Hong Ki An, Shanhua Zhang, Seyed Mohammadreza Ghadiri

**Affiliations:** 1 School of Civil Engineering, Universiti Sains Malaysia, Engineering Campus, 14300 Nibong Tebal, Pulau Pinang Malaysia; 2 School of Traffic and Transportation, Shijiazhuang Tiedao University, Shijiazhuang, 050047 China; 3 Department of Digital Equipment, Jiangsu Vocational College of Electronics and Information, Huai’an, China; 4 Swiss Information and Management Institute, 6340 Baar, Switzerland

**Keywords:** Optimized GM(0,N), Queue length, Metered roundabout, Combined exponential and trigonometric transformations, Particle swarm optimization, Engineering, Mathematics and computing, Physics

## Abstract

This study proposes an optimized GM(0,N) model that integrates entry traffic volume, conflicting flow, and signal timing (green and red times) for predicting queue lengths at metered roundabouts. While conventional GM models have the advantage of requiring few samples and offering high prediction accuracy, they can lead to prediction errors as they do not consider the influence of other factors on time series. To enhance prediction accuracy, the proposed model transforms the original sequence by combining exponential and trigonometric functions to overcome the limitations of single transformation functions in processing original sequences and the difficulty of fixed transformation functions adapting to various data types. Optimal parameters are then determined using PSO. This model was validated using real-world data obtained from metered roundabouts in Adelaide, Australia. Compared to An’s model, as well as the GM(1,1), GM(1,N), and conventional GM(0,N) models, the proposed method demonstrated superior accuracy across MRE, RMSE, MAE, and box plot analyses. These results support the model’s applicability for managing unbalanced roundabout traffic and for effective detector placement.

## Introduction

Roundabouts, where entering vehicles yield to circulating traffic, offer advantages over signalized intersections in terms of increased intersection capacity, enhanced safety, and reduced vehicular emissions. As a result, they have been widely adopted and actively operated in countries such as Australia, the United States, and across Europe, and are increasingly recognized as a new type of intersection control device worldwide^1–3^.

However, when unexpected increases in traffic volume lead to congestion at roundabouts, signalized roundabouts emerge as a viable alternative^4–8^. As shown in Table 1, signalized roundabouts can be classified into six control types, depending on traffic flow management, operational hours, and the number of controlled approaches^1^.

**Table 1:** Types of signal roundabout.

Type	Control method	Description
Traffic flow control	Direct control	Entering traffic flow and circulatory traffic flow are controlled by signals
	Indirect control	Entering traffic flows only are controlled by signals
Operation time	Full time control	Signal is operated for 24 h
	Part time control	Signal is activated by time of day or by detectors
Number of approaches controlled	Full control	All approaches are controlled by signals
	Partial control	Approaches are controlled by signals while the remaining approaches operate under give way control

In particular, during peak periods, unbalanced traffic conditions often arise when traffic entering specific approaches is unevenly distributed, leading to prolonged delays on certain approaches. To mitigate this issue, studies have examined the installation of detectors on the major approaches and traffic signals on the minor approaches that interfere with the entry to the major road. This strategy, referred to as Indirect, Part-time, or Partial control, has been investigated as an effective control method.

**Fig. 1:**
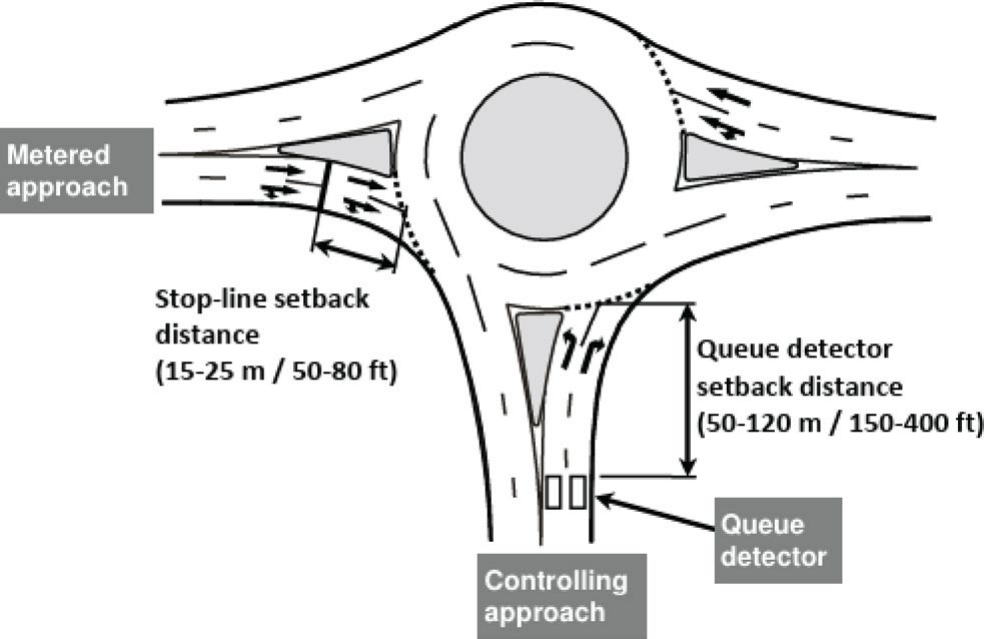
A metered roundabout concepts^4^.

A metered roundabout operates by installing detectors on the main (controlling) approach, where long queues typically form during peak periods, and traffic signals on the approach that most interferes with entry to the main approach, as illustrated in Fig. 1. This configuration helps to balance traffic under unbalanced conditions^4^. For example, if a detector is installed at a distance of 50 m from the stop line on the controlling approach, the signal on the metered approach will turn red when the queue on the controlling approach exceeds 50 m, thereby preventing further entry from the metered approach. In this scenario, vehicles on the controlling approach can easily secure sufficient gap times. All other approaches continue to operate under normal roundabout conditions. If the queue on the controlling approach does not reach 50 m, the intersection functions as a conventional roundabout. Hence, in metered roundabouts, the location of the detector is closely related to queue formation and directly affects overall roundabout performance^9^.

Queue length at intersections is one of the most critical performance measures for the design and operation of traffic signals^10–15^. In particular, predicting queue lengths at metered roundabouts plays a key role in determining the optimal placement of detectors, making it an essential aspect to study when converting a conventional roundabout into a metered roundabout. However, unlike conventional signalized intersections, research on queue lengths at metered roundabouts remains limited. At signalized intersections, vehicle entry on all approaches is governed by traffic signals, whereas at metered roundabouts, the metered approach is controlled by a signal while the controlling approach and other approaches operate according to the characteristics of a standard roundabout. Consequently, different modeling approaches are required. Among the influencing factors, the rotating vehicles that interfere with the entry of other approaches are particularly significant^3,9,16^.

Recently, various studies have actively investigated queue lengths at intersections^17^. The first category consists of studies that utilize conventional queueing theory, which assumes Poisson arrival processes and incorporates signal cycles and saturation flow rates, or the Webster model. This method is grounded in the theory that queue lengths are derived from cumulative arrivals and departures, but it faces difficulties in computing the spatial distribution of queues. Second, studies based on shockwave theory have been carried out. This approach predicts queue lengths for each signal cycle by calculating the propagation speed of shockwaves resulting from changes in traffic flow states. Lastly, queue length prediction using Deep Reinforcement Learning (DRL) has gained popularity due to advances in artificial intelligence algorithms. However, it suffers from slow convergence caused by inefficient selection of training samples^18^. In addition, the majority of studies have focused on queue length at conventional signalized intersections, while research on queue length at metered roundabouts remains limited.

After several decades of development, grey system theory has been widely applied in the field of transportation^19,20^. The GM(1,1) model, an important prediction approach within grey system theory, offers advantages such as requiring only a small sample size and providing high forecasting accuracy^21^. However, the traditional GM(1,1) model does not account for the influence of other factors on the time series, which limits its applicability when dealing with time series affected by multiple factors. The GM(1,N) model^22^ addresses this limitation, but issues such as the nonequivalence between the grey differential equation and the whitening equation, as well as computational deviations in background value sequences, inevitably affect its prediction accuracy.

GM(0,N) models can avoid the aforementioned disadvantages as they do not involve differential equations or whitening equations. Nevertheless, the adaptability of the original sequence to the model is a problem that exists in all grey prediction models. Liu et al.^23^ proposed several transformation methods, including logarithmic transformation, square root transformation, power function transformation, scalar multiplication transformation, exponential transformation, trigonometric transformation, and inverse trigonometric transformation. While these approaches can improve the applicability of the original sequence to some extent, they suffer from the limited processing capability of a single transformation function and the difficulty of generalizing a fixed transformation function to various types of data sequences. To address these issues, this study proposes a new composite transformation function and incorporates adjustable parameters into the function to enhance its adaptability to various types of data.

Therefore, this study aims to propose an improved GM(0,N) model based on the Grey Model (GM) for predicting the queue length of each approach at metered roundabouts, which is capable of making predictions with incomplete or small-scale datasets. The GM(0,N) model provides a framework similar to a static regression model, revealing how multiple variables influence queue length. To further enhance the predictive accuracy of the GM(0,N) model, the original queue length series is transformed using a combined function of sine and exponential functions to obtain a new sequence. The GM(0,N) model is then constructed with the transformed sequence to generate prediction results, which are subsequently restored to produce the final forecast. In order to make the model applicable to different data types, the combined transformation function contains two unknown parameters. To improve both predictive accuracy and parameter estimation, the optimal values of these two parameters are determined using the Particle Swarm Optimization (PSO) algorithm.

The optimized GM(0,N) model was evaluated against five datasets: conventional GM(1,1), GM(1,N), GM(0,N), field observations, and metered roundabout queue lengths predicted by An. An analysis using the Mean Relative Error (MRE), Root Mean Square Error (RMSE), and Mean Absolute Error (MAE) was conducted to assess the accuracy of predictions against actual measurements. The study utilized data from the SCATS system at the Old Belair Road roundabout in Adelaide, Australia, operating under a metered system during the morning peak, supplemented by drone-based measurements of queue lengths.

## Literature review

As discussed earlier, a wide range of studies has been conducted on predicting queue lengths at intersections. Among those considering arrival flow, saturation flow, and signal timing, many have focused on improving classical queuing theory or the Webster model. Verma et al.^24^ examined queue length prediction at intersections in India under mixed traffic conditions. Since the M/D/1 model, which assumes deterministic service times and random arrivals, was unsuitable for Indian traffic conditions, an improved M/M/N model incorporating the concept of probably-first-in–probably-first-out was proposed. An analysis of five intersections revealed that the proposed model reduced the error rate by 20% compared with the traditional Webster model. Liu et al.^25^ applied spatial interpolation correction to the statistical characteristics of vehicle arrivals and departures at signalized intersections and employed a Markov model to dynamically predict major queuing points. A case study at the Hongkong–Fuzhou intersection in Qingdao City showed a 3% reduction in error compared with the conventional Webster model. More recently, Jithender and Mehar^26^ demonstrated that various queue prediction models failed to match field observations and developed a multivariate model that incorporated the distribution of two-wheelers as a key variable. However, its accuracy improved only when the proportion of two-wheelers exceeded 30% and traffic volume approached saturation, indicating certain limitations.

Research on queue length prediction at roundabouts using the Webster model was conducted by Bie et al.^27^. This study focused on a fully signal-controlled roundabout and considered the influence of left-turning vehicles on other approaches when calculating queue lengths. The proposed model was tested at the Zhonghua roundabout in Guizhou Province, China, and reported maximum and minimum prediction errors of 13.6% and 3.3%, respectively. Similarly, Macioszek and Iwanowicz^11^ investigated queue length prediction at conventional roundabouts by considering both the starting-up flow rate and the maximum departure flow rate. However, it did not account for the influence of circulating vehicles within the roundabout, which is a critical factor affecting queue length. The proposed method achieved a correlation coefficient of 0.877 and a determination coefficient of 0.769.

A variety of studies have also been conducted based on shockwave theory. Li et al.^10^ applied the Lighthill–Whitham–Richards (LWR) shockwave theory together with the Robertson model to perform lane-based queue length prediction at 5-s intervals. The model achieved an Root Mean Square Error (RMSE) of 2.33 vehicles, an Mean Absolute Error (MAE) of 1.82 vehicles, and a Mean Absolute Percentage Error (MAPE) of 16.12%. Liu et al.^28^ proposed a Markov model that considered average traffic volume, historical queue lengths, and arrival vehicle data as variables, demonstrating higher accuracy compared with conventional models. Gao et al.^12^ combined shockwave theory with deep learning techniques to estimate queue lengths at intersections, yielding an average prediction error of approximately 10%. Horváth and Tettamanti^29^ accounted for green time within each cycle and vehicle trajectories using kinematic analysis. Simulation results based on a 60-s cycle length showed an MAE of 2.09 vehicles. Ferencz and Zöldy^30^ applied machine learning and the Kalman filter algorithm to estimate queue lengths and validated the model using the SUMO network. While earlier studies simulated the effects of stop-and-go waves on queue formation, this study estimated queue lengths by calculating speeds from distance traveled in each time unit through the Kalman filter, resulting in improved accuracy over traditional models. Furthermore, Shafik and Rakha^31^ predicted turning movements at signalized intersections and applied a Kalman filter to estimate queue lengths, achieving a 32.8% improvement in prediction accuracy.

In recent years, increasing attention has been directed toward queue length prediction using artificial intelligence algorithms. An et al.^32^ employed an Adaptive Neuro-Fuzzy Inference System algorithm to predict queue lengths at each approach of a roundabout, and model validation was performed using RMSE and R² metrics. Abewickrema et al.^33^ used a multilayer feedforward neural network to predict queue lengths at 2-s intervals for the Brisbane M5008 intersection and reported that it outperformed the conventional Kalman filter–based approach. Rahman and Hasan^34^ applied a deep learning framework based on the Long Short-Term Memory (LSTM) concept to estimate queue lengths at signalized intersections. A comparative analysis was conducted on 11 intersections in Orlando against Support Vector Regression and K-Nearest Neighbors methods. Results demonstrated that the LSTM-based model consistently yielded lower RMSE and MSE values across all intersections. More recently, Cai and Wei^35^ introduced a model employing Double Q-learning within a Deep Reinforcement Learning (DRL) framework. Under an 80-s fixed-time control signal cycle, the proposed model achieved prediction accuracy improvements of 39.52%, 32.83%, and 15.76% compared with Max-Pressure, Dueling Double Deep Q-Network, and baseline schemes, respectively. Chen et al.^36^ proposed a system called Traffic Talk that utilizes deep learning techniques to determine optimal signal timings under mixed-traffic conditions. This method converts collected traffic data into vehicle density maps and predicts queue lengths by reflecting the spatial distribution of different vehicle types. Gu et al.^37^ also proposed a queue-length prediction method based on the deep-learning concept of multilayer perceptrons, employing a Convolutional Neural Network–Long Short-Term Memory–Attention architecture. They introduced a new approach to determine the time point at which the queue dissipates during the green phase under Poisson-distributed vehicle arrivals at signalized intersections. Klaykul et al.^38^ presented a method that integrates deep learning with multi-objective optimization to predict queue lengths at highway toll plazas. Using real-time data, they employed a Gated Recurrent Unit model to estimate the average queue length without disrupting traffic flow.

Most existing studies have been confined to predicting queue lengths at signalized intersections, while research on queue lengths at roundabouts, particularly at metered roundabouts, remains very limited. A study conducted by An et al.^16^ considered variables such as arrival traffic volume, circulating flow, detector location, green and red times, headway, and occupancy time at detectors to predict queue lengths for each approach.

As reviewed above, most existing studies have been limited to queue length prediction at signalized intersections, whereas research on roundabouts—particularly metered roundabouts—remains very scarce. A study conducted by An et al.^16^ estimated queue lengths at 5-minute intervals by considering variables such as arrival flow, circulating flow, detector location, green and red times, headway, and detector occupancy time. The queue length equations for each approach were presented in Eqs. (1)–(3). Based on An’s model, the present study incorporates variables including traffic volume, conflicting flow, and signal timings (green and red) in the prediction of queue lengths.

Although this study proposed a method for estimating the queue length at each approach of metered roundabouts, the root mean value ranging from 0.71 to 0.72 indicates that its accuracy remains somewhat limited.

- Controlling approach

1 $${Q_c}=\frac{{\left( {\frac{{{P_{green}}}}{T} \times \frac{{\frac{{{V_i}}}{{NL}}}}{T} \times \frac{{\frac{{V{C_i}}}{{NL}}}}{T} \times D{L_C} \times P{T_C} \times VS} \right)}}{{D{L_M} \times P{T_M}}} \times \alpha$$

- Metered approach

2 $${Q_m}=\frac{{\left( {\frac{{{P_{red}}}}{T} \times \frac{{\frac{{{V_i}}}{{NL}}}}{T} \times \frac{{\frac{{V{C_i}}}{{NL}}}}{T} \times D{L_M} \times P{T_M} \times VS} \right)}}{{D{L_C} \times P{T_C}}} \times ~\beta$$

- Other approaches

3 $${Q_o}=\frac{{\left( {\frac{{{P_{green}}}}{T} \times \frac{{\frac{{{V_i}}}{{NL}}}}{T} \times \frac{{\frac{{V{C_i}}}{{NL}}}}{T} \times D{L_C} \times P{T_C} \times VS} \right)}}{{D{L_M} \times P{T_M}}} \times \gamma$$

where, ${Q_c}$, ${Q_m}$ and ${Q_o}$ represent queue length on controlling, metered and other approaches (m), ${P_{green}}$ and ${P_{red}}$ are signal green time and red time (s), ${V_i}$ is arrival volume on *i* approach (pcu), ${V_{ci}}$ is conflict volume for *i* approach (pcu), $D{L_C}$and $D{L_M}$are detector location of controlling and metered approach (m), $P{T_C}$ and $P{T_C}$ are vehicle presence time on detector (s), VS is vehicle space (m), *T* is survey time (s), *NL* is number of lane, and *α*, *β*, *γ* are calibration constant.

## Research methods

### Research procedure

**Fig. 2:**
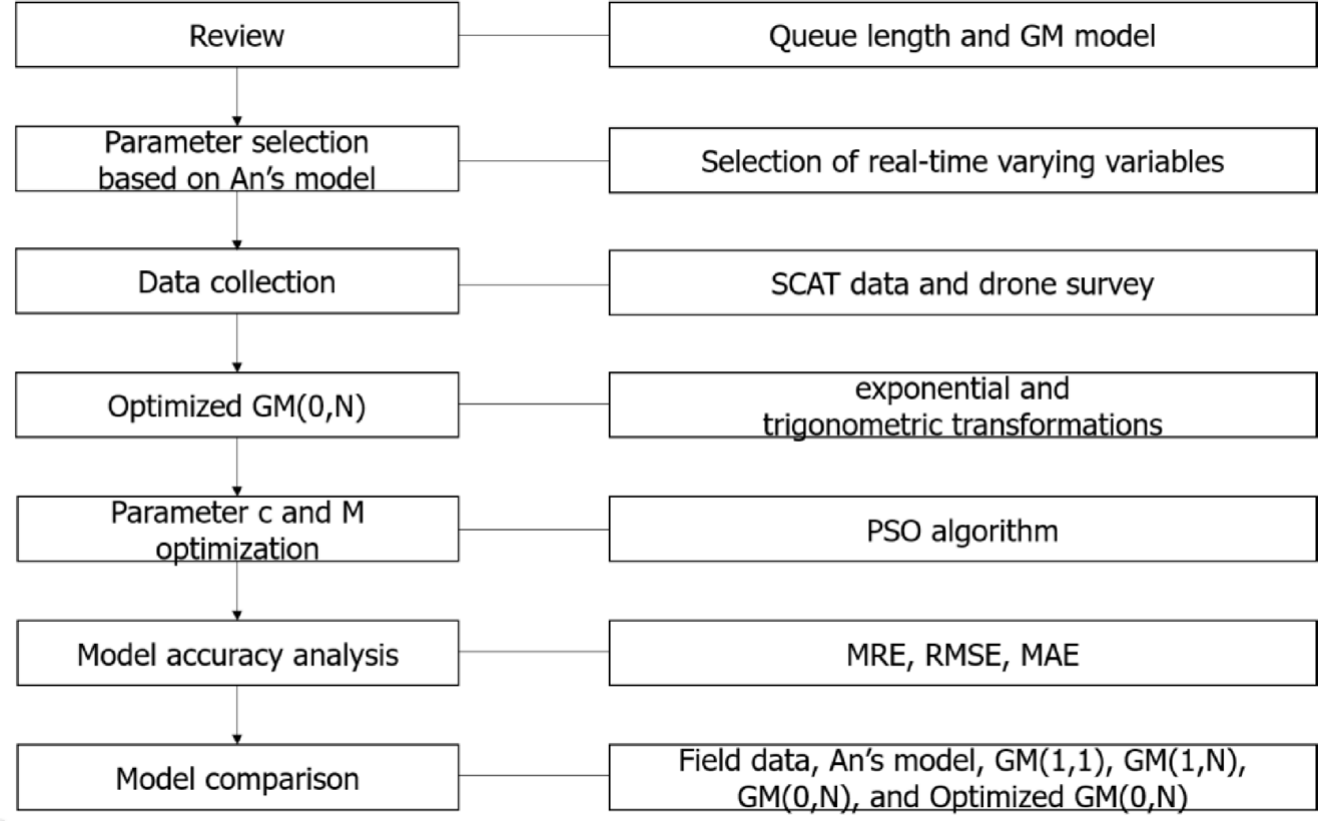
Research flow.

This study aims to develop a model for estimating the queue length of each approach at metered roundabouts, with the overall research framework illustrated in Fig. 2. Field data for model development were collected at the Old Belair Road roundabout in Adelaide, Australia. A review of existing queue length estimation studies was then conducted, along with an examination of the theoretical foundations of the Grey Model (GM). Building upon An’s model, real-time varying variables were identified, and exponential as well as trigonometric transformations were incorporated within the GM(0,N) framework. Subsequently, PSO was applied to determine the optimal values of the parameters *c* and *M*. The precision of the model was verified through MRE, RMSE, and MAE analysis, after which the proposed GM(0,N) model was benchmarked against existing methods namely GM(1,1), GM(1,N), GM(0,N), An’s model, and field measurements to evaluate predictive accuracy.

### Grey relational analysis

Grey Relational Analysis (GRA) is a quantitative approach used to evaluate the dynamic behavior of a system by measuring the geometric similarity between a reference sequence and several comparative sequences. This similarity indicates the strength of their relationships^39^. GRA has been extensively applied in transportation research^40^.

This study predicts the queue length at each entry of a roundabout based on four influencing factors: entry traffic volume, conflicting traffic volume, green signal duration, and red signal duration. To examine how these factors are related to queue length, GRA is employed to assess the degree of association between them.

#### Calculation of grey relational analysis

1. Dimensionless transformation of variables

In grey relational analysis, the indicators within a system often differ in units and physical meaning, resulting in inconsistent dimensions across the dataset. To enable valid comparisons and obtain reliable results, a normalization method is applied to transform both queue length data and influencing factor data into a dimensionless form. As shown in Eq. (4), this process scales all data values into the interval [0,1].

4 $${\mathrm{y=}}\left( {{{\mathrm{y}}_{{\mathrm{max}}}} - {{\mathrm{y}}_{{\mathrm{min}}}}} \right) \cdot \left( {{\mathrm{z}} - {{\mathrm{z}}_{{\mathrm{min}}}}} \right)/\left( {{{\mathrm{z}}_{{\mathrm{max}}}} - {{\mathrm{z}}_{{\mathrm{min}}}}} \right)+{{\mathrm{y}}_{{\mathrm{min}}}}$$

In this Eq. (4), *z* represents the raw data to be normalized, forming the sequence *Z*. The terms ${{\mathrm{z}}_{{\mathrm{max}}}}$​ and ${{\mathrm{z}}_{{\mathrm{min}}}}$​ denote the maximum and minimum values within the sample sequence, respectively. The variable *y* refers to the normalized value after preprocessing, forming the sequence *Y*. By definition, the maximum value of the normalized sequence ${{\mathrm{y}}_{{\mathrm{max}}}}$ is set to 1, and the minimum value ${{\mathrm{y}}_{{\mathrm{min}}}}$ is set to 0. If the original sequence needs to be transformed into a different interval, this can be achieved by adjusting the parameters ${{\mathrm{y}}_{{\mathrm{min}}}}$​ and ${{\mathrm{y}}_{{\mathrm{max}}}}$​.

(2) Calculation of grey relational coefficients and grey relational grades

The grey relational coefficient is presented in Eq. (5):

5 $${{\mathrm{\boldsymbol{\updelta}}}_{\mathrm{i}}}\left( {\mathrm{k}} \right){\mathrm{=}}\frac{{{\mathrm{mi}}{{\mathrm{n}}_{\mathrm{i}}}{\mathrm{mi}}{{\mathrm{n}}_{\mathrm{k}}}\left| {{\mathrm{y}}_{{\mathrm{0}}}^{\prime }\left( {\mathrm{k}} \right) - {\mathrm{y}}_{{\mathrm{i}}}^{\prime }\left( {\mathrm{k}} \right)} \right|{\mathrm{+\boldsymbol{\uprho}}} \cdot {\mathrm{ma}}{{\mathrm{x}}_{\mathrm{i}}}{\mathrm{ma}}{{\mathrm{x}}_{\mathrm{k}}}\left| {{\mathrm{y}}_{{\mathrm{0}}}^{\prime }\left( {\mathrm{k}} \right) - {\mathrm{y}}_{{\mathrm{i}}}^{\prime }\left( {\mathrm{k}} \right)} \right|}}{{\left| {{\mathrm{y}}_{{\mathrm{0}}}^{\prime }\left( {\mathrm{k}} \right) - {\mathrm{y}}_{{\mathrm{i}}}^{\prime }\left( {\mathrm{k}} \right)} \right|{\mathrm{+\boldsymbol{\uprho}}} \cdot {\mathrm{ma}}{{\mathrm{x}}_{\mathrm{i}}}{\mathrm{ma}}{{\mathrm{x}}_{\mathrm{k}}}\left| {{\mathrm{y}}_{{\mathrm{0}}}^{\prime }\left( {\mathrm{k}} \right) - {\mathrm{y}}_{{\mathrm{i}}}^{\prime }\left( {\mathrm{k}} \right)} \right|}}$$

In Eq. (5), ${{\mathrm{\boldsymbol{\updelta}}}_{\mathrm{i}}}\left( {\mathrm{k}} \right)$ denotes the grey relational coefficient. The term $\left| {{\mathrm{y}}_{{\mathrm{0}}}^{{{\prime }}}\left( {\mathrm{k}} \right){\mathrm{-y}}_{{\mathrm{i}}}^{{{\prime }}}\left( {\mathrm{k}} \right)} \right|$ represents the absolute difference (referred to as the *local deviation*) between the reference sequence ${{\mathrm{Y}}_{\mathrm{0}}}$​ and the comparison sequence ${{\mathrm{Y}}_{\mathrm{i}}}$​ at the *k*-th sample point; ${\mathrm{mi}}{{\mathrm{n}}_{\mathrm{i}}}{\mathrm{mi}}{{\mathrm{n}}_{\mathrm{k}}}\left| {{\mathrm{y}}_{{\mathrm{0}}}^{{{\prime }}}\left( {\mathrm{k}} \right){\mathrm{-y}}_{{\mathrm{i}}}^{{{\prime }}}\left( {\mathrm{k}} \right)} \right|$ and ${\mathrm{ma}}{{\mathrm{x}}_{\mathrm{i}}}{\mathrm{ma}}{{\mathrm{x}}_{\mathrm{k}}}\left| {{\mathrm{y}}_{{\mathrm{0}}}^{{{\prime }}}\left( {\mathrm{k}} \right){\mathrm{-y}}_{{\mathrm{i}}}^{{{\prime }}}\left( {\mathrm{k}} \right)} \right|$ indicate the minimum and maximum deviations across all sequences, respectively. The parameter ${\mathrm{\boldsymbol{\uprho}}}$ is the distinguishing coefficient, generally set to ${\mathrm{\boldsymbol{\uprho}}}$ = 0.5.

The grey relational grade ${{\mathrm{r}}_{\mathrm{i}}}$​, which reflects the overall correlation between the reference sequence and a comparison sequence, is then calculated using the following Eq. (6):

6 $${{\mathrm{r}}_{\mathrm{i}}}{\mathrm{=}}\frac{{\mathrm{1}}}{{\mathrm{n}}}\mathop \sum \limits_{{{\mathrm{k=1}}}}^{{\mathrm{n}}} {{\mathrm{\boldsymbol{\updelta}}}_{\mathrm{i}}}\left( {\mathrm{k}} \right)$$

The grey relational grade ${{\mathrm{r}}_{\mathrm{i}}}$​ is obtained by averaging the grey relational coefficients ${{\mathrm{\boldsymbol{\updelta}}}_{\mathrm{i}}}\left( {\mathrm{k}} \right)$ over all sample points. Its value ranges between [0,1]. A value closer to 1 indicates that the comparison sequence exhibits a trend more similar to the reference sequence, implying a stronger correlation; conversely, a value closer to 0 suggests weaker similarity and thus a lower degree of correlation.

#### Results of grey relational analysis

**Fig. 3:**
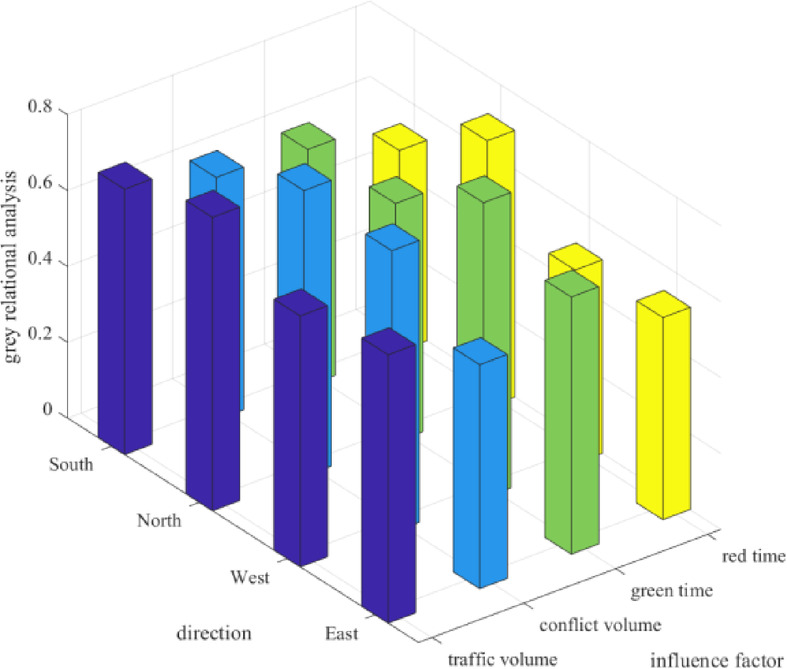
Grey relational grades between queue length and influencing factors.

The intersection under study consists of four entry approaches. Using the above equations, the grey relational grades between queue length and the four influencing factors, namely entry traffic volume, conflicting traffic volume, green signal duration, and red signal duration, were calculated for each approach. The results are presented in Fig. 3.

According to the three-dimensional bar chart, the X-axis represents the four influencing factors, the Y-axis denotes the four entry approaches of the intersection, and the Z-axis corresponds to the grey relational grade. All grey relational grades are greater than 0.5, indicating that, regardless of direction, each of the four factors is correlated with queue length and can thus be considered as a valid input variable for modeling. Among the factors, entry traffic volume, conflicting traffic volume, and green signal duration exhibit relatively higher correlations with queue length. Except for the East approach, where the correlation between conflicting traffic volume and queue length is below 0.6, all other correlations exceed 0.6. In contrast, red signal duration shows weaker correlations, with only the North approach reaching 0.7, while all other approaches remain below 0.6.

### Prediction of queue length at intersections based on grey models

#### Prediction using three grey models

1. GM(1,1) model

The GM(1,1) model, formally known as the First-Order One-Variable Grey Forecasting Model, is one of the most fundamental and widely applied models within the grey system theory framework. This theory was proposed by Chinese scholar Deng in the late 1970s to early 1980s, originating from the limitations of conventional system analysis methods in addressing problems characterized by “partially known and partially unknown” information. In reality, many systems are neither “fully known” nor “completely unknown”; such systems were defined by Deng as “grey systems,” for which traditional methods often fail to provide effective modeling.

To address this issue, Deng formally introduced grey system theory in his 1982 publication Grey Control Systems^41^. The core idea is to “extract and process the partially known information to reduce the system’s ‘greyness,’ thereby establishing concise and reliable models for predicting or controlling system behavior.” This theory broke through the traditional dichotomy of system classification (“black or white”) and provided a novel analytical tool for scenarios characterized by small samples, limited information, and uncertainty^42^. Moreover, the GM(1,1) model has been extensively applied in the field of transportation^41^.

When applying the GM(1,1) model to predict queue length at intersections, external influencing factors are not considered. Instead, the model relies solely on historical data to capture and forecast future trends. Taking the queue length in one approach of a roundabout as an example, the original dataset consists of 60 time-series observations of queue length. The first 50 observations are used to calibrate the GM(1,1) model and derive the forecasting equation, which is then applied to predict the remaining 10 samples.

(2) GM(1,N) model

In the GM(1,1) model, the prediction of intersection queue length does not take influencing factors into account, which reduces the reliability of the results. In contrast, the GM(1,N) model, as another type of grey prediction model, incorporates multiple influencing factors. Therefore, compared with the GM(1,1) model, the GM(1,N) model is more suitable for predicting queue length at intersections. The GM(1,N) model is a multivariable prediction model within grey system theory. Here, the first “1” denotes a first-order differential equation, while “*N*” indicates the inclusion of *N* variables, with one serving as the dependent variable and the remaining *N* − 1 as independent variables. The model is primarily used to analyze the dynamic relationships between the dependent variable and multiple independent variables, and to perform prediction^43^.

Using the GM(1,N) model to forecast intersection queue length while incorporating all four influencing factors—Taking the queue length in one approach of a roundabout as an example—let ${\mathrm{X}}_{{\mathrm{1}}}^{{{\mathrm{(0)}}}}$​ denote the queue-length sequence, and let ${\mathrm{X}}_{{\mathrm{2}}}^{{{\mathrm{(0)}}}}{\mathrm{,~X}}_{{\mathrm{3}}}^{{{\mathrm{(0)}}}}{\mathrm{,~X}}_{{\mathrm{4}}}^{{{\mathrm{(0)}}}}{\mathrm{,~X}}_{{\mathrm{5}}}^{{{\mathrm{(0)}}}}$​ denote the sequences of entry traffic volume, conflicting traffic volume, green signal duration, and red signal duration, respectively. The original dataset comprises 60 time-series observations. The first 50 observations are used to fit the GM(1,N) model and obtain the prediction equation, and the fitting errors for these 50 points are evaluated. The resulting prediction equation is then used to forecast the final 10 observations, and the prediction errors for these 10 points are assessed.

(3) GM(0,N) model

Based on the GM(1,N) model, the queue length at intersections can be predicted by incorporating multiple influencing factors. However, the GM(1,N) model is relatively complex to construct, and its modeling process has certain limitations that reduce prediction accuracy. Specifically, the GM(1,N) model estimates the parameters ${\mathrm{a,}}{{\mathrm{u}}_{\mathrm{2}}}{\mathrm{,}}{{\mathrm{u}}_{\mathrm{3}}}{\mathrm{,}}...{\mathrm{,}}{{\mathrm{u}}_{\mathrm{N}}}$ through a grey differential equation, then derives the time series expression from the whitening equation, and substitutes the parameters into the whitening equation for prediction. Since the grey differential equation and the whitening equation are not equivalent, discrepancies arise. Moreover, the calculation of the background value sequence ${\mathrm{z}}_{{\mathrm{1}}}^{{{\mathrm{(1)}}}}\left( {\mathrm{k}} \right)$ is prone to significant deviations. These issues constitute the primary sources of prediction error in the GM(1,N) model.

In contrast, the GM(0,N) model avoids these drawbacks, as it does not involve the coupling between the differential equation and the whitening equation, nor does it require the calculation of a background value sequence. Consequently, its modeling process is simpler. Unlike the dynamic analysis of the GM(1,N) model, the GM(0,N) model is essentially a “zero-order” model (i.e., without differential equations and without explicit consideration of temporal trends). It directly constructs linear relationships through cumulative data, emphasizing the “static quantification of factor associations.” The formulation is concise and easier to interpret.

The dependent variable sequence is denoted as ${\mathrm{X}}_{{\mathrm{1}}}^{{{\mathrm{(0)}}}}$, while the *N* − 1 independent variable sequences are represented as ${\mathrm{X}}_{{\mathrm{2}}}^{{{\mathrm{(0)}}}}{\mathrm{,~X}}_{{\mathrm{3}}}^{{{\mathrm{(0)}}}}{\text{,~ \ldots ,~X}}_{{\mathrm{N}}}^{{{\mathrm{(0)}}}}$. The original sequences can be expressed as follows:

$$\begin{array}{*{20}l} {X_{1}^{{\left( 0 \right)}} = \left\{ {x_{1}^{{\left( 0 \right)}} \left( 1 \right),~x_{1}^{{\left( 0 \right)}} \left( 2 \right), \ldots ,x_{1}^{{\left( 0 \right)}} \left( n \right)} \right\},~~} \hfill \\ {X_{2}^{{\left( 0 \right)}} = \left\{ {x_{2}^{{\left( 0 \right)}} \left( 1 \right),~x_{2}^{{\left( 0 \right)}} \left( 2 \right), \ldots ,x_{2}^{{\left( 0 \right)}} \left( n \right)} \right\},~} \hfill \\ \ldots \hfill \\ {X_{N}^{{\left( 0 \right)}} = \left\{ {x_{N}^{{\left( 0 \right)}} \left( 1 \right),~x_{N}^{{\left( 0 \right)}} \left( 2 \right), \ldots ,x_{N}^{{\left( 0 \right)}} \left( n \right)} \right\},} \hfill \\ \end{array}$$

where *n* denotes the length of the data sequence. By performing the first-order accumulated generating operation (AGO) on each sequence ${\mathrm{X}}_{{\mathrm{i}}}^{{{\mathrm{(0)}}}}$​, the transformed sequences are obtained as:

7 $$X_{i}^{{\left( 1 \right)}}=\{ x_{i}^{{\left( 0 \right)}}\left( 1 \right),~x_{i}^{{\left( 0 \right)}}\left( 2 \right), \ldots ,x_{i}^{{\left( 0 \right)}}\left( n \right),~i=1,2,3, \ldots ,n$$

The main computational process is defined as follows:

8 $${\mathrm{x}}_{{\mathrm{i}}}^{{{\mathrm{(1)}}}}\left( k \right){\mathrm{=}}\mathop \sum \limits_{{{\mathrm{i=1}}}}^{{\mathrm{k}}} {\mathrm{x}}_{{\mathrm{i}}}^{{{\mathrm{(0)}}}}\left( k \right){\text{,~k=1,2,3, \ldots ,n}}$$

Subsequently, a linear model is constructed. The core of the GM(0,N) model lies in establishing a linear expression between the dependent variable and the independent variables:

9 $$\widehat{{\mathrm{x}}}_{{\mathrm{1}}}^{{\left( {\mathrm{1}} \right)}} \left( {\mathrm{k}} \right){\text{ = b}}_{{\mathrm{0}}} {\text{ + }}\sum\limits_{{{\text{i = 2}}}}^{{\mathrm{N}}} {{\mathrm{b}}_{{\mathrm{i}}} } {\mathrm{x}}_{{\mathrm{i}}}^{{{\mathrm{(1)}}}} \left( {\mathrm{k}} \right){\mathrm{,}}\;{\text{k = 1}},{\mathrm{2}},{\mathrm{3}}, \ldots ,{\mathrm{n}}$$

where, ${{\hat{\mathrm{x}}}}_{{\mathrm{1}}}^{{\left( {\mathrm{1}} \right)}} \left( {\mathrm{k}} \right)$ is the estimated value of the accumulated sequence of the dependent variable, ${{\mathrm{b}}_{\mathrm{0}}}$​ is the constant term (intercept), and ${{\mathrm{b}}_{\mathrm{i}}}{\text{~(i=2, \ldots ,N)}}$ represent the regression coefficients. The parameters ${{\mathrm{b}}_{\mathrm{0}}}$​ and ${{\mathrm{b}}_{\mathrm{i}}}$​ can be obtained using the least squares method, as described in Eq. (10):

10 $${\left( {{{\mathrm{b}}_{\mathrm{0}}}{\mathrm{,}}{{\mathrm{b}}_{\mathrm{2}}}{\mathrm{,}}{{\mathrm{b}}_{\mathrm{3}}}{\mathrm{,}}...{\mathrm{,}}{{\mathrm{b}}_{\mathrm{N}}}} \right)^{\mathrm{T}}}{\mathrm{=}}{\left( {{{\mathrm{B}}^{\mathrm{T}}}{\mathrm{B}}} \right)^{{\mathrm{-1}}}}{{\mathrm{B}}^{\mathrm{T}}}{\mathrm{Y}}$$

where, $${\mathrm{B=}}\left[ {\begin{array}{*{20}{c}} {\mathrm{1}}&{{\mathrm{x}}_{{\mathrm{2}}}^{{{\mathrm{(1)}}}}{\mathrm{(1)}}}&{...}&{{\mathrm{x}}_{{\mathrm{N}}}^{{{\mathrm{(1)}}}}{\mathrm{(1)}}} \\ {\mathrm{1}}&{{\mathrm{x}}_{{\mathrm{2}}}^{{{\mathrm{(1)}}}}{\mathrm{(2)}}}&{...}&{{\mathrm{x}}_{{\mathrm{N}}}^{{{\mathrm{(1)}}}}{\mathrm{(2)}}} \\ {...}&{...}&{...}&{...} \\ {\mathrm{1}}&{{\mathrm{x}}_{{\mathrm{2}}}^{{{\mathrm{(1)}}}}{\mathrm{(n)}}}&{...}&{{\mathrm{x}}_{{\mathrm{N}}}^{{{\mathrm{(1)}}}}{\mathrm{(n)}}} \end{array}} \right]$$, $${\mathrm{Y=}}\left[ {\begin{array}{*{20}{c}} {{\mathrm{x}}_{{\mathrm{1}}}^{{{\mathrm{(0)}}}}{\mathrm{(1)}}} \\ {{\mathrm{x}}_{{\mathrm{1}}}^{{{\mathrm{(0)}}}}{\mathrm{(2)}}} \\ {...} \\ {{\mathrm{x}}_{{\mathrm{1}}}^{{{\mathrm{(0)}}}}{\mathrm{(n)}}} \end{array}} \right]$$. After estimating the parameter values, they are substituted into Eq. (9) and then subjected to the inverse accumulation process. The restoration is expressed as:

11 $${{\hat {\mathrm{x}}}}_{{\mathrm{1}}}^{{\left( {\mathrm{0}} \right)}}\left( {\mathrm{k}} \right){{=\hat {\mathrm{x}}}}_{{\mathrm{1}}}^{{\left( {\mathrm{1}} \right)}}\left( {\mathrm{k}} \right) - {{\hat {\mathrm{x}}}}_{{\mathrm{1}}}^{{\left( {\mathrm{1}} \right)}}\left( {{\mathrm{k}} - {\mathrm{1}}} \right){\mathrm{,}}$$

Which yields the prediction formula for the original sequence:$~\widehat{{\mathrm{x}}}_{{\mathrm{1}}}^{{\left( {\mathrm{0}} \right)}} \left( {\mathrm{k}} \right),~{\mathrm{k}}~{\text{ = }}~{\mathrm{1}},~{\mathrm{2}},~{\mathrm{3}},~ \ldots ,~{\mathrm{n}}$

The GM(0,N) model is employed to predict intersection queue length, with the sample data from a selected approach of a roundabout used as an illustrative example. Let ${\mathrm{X}}_{{\mathrm{1}}}^{{{\mathrm{(0)}}}}$​ denote the queue-length sequence at the intersection; ${\mathrm{X}}_{{\mathrm{2}}}^{{{\mathrm{(0)}}}}{\mathrm{,~X}}_{{\mathrm{3}}}^{{{\mathrm{(0)}}}}{\mathrm{,~X}}_{{\mathrm{4}}}^{{{\mathrm{(0)}}}}{\mathrm{,X}}_{{\mathrm{5}}}^{{{\mathrm{(0)}}}}$ ​ denote the sequences of upstream arrival flow, conflicting flow, green-time, and red-time, respectively. A total of 60 original observations are available. The first 50 observations are used to fit the model and derive the prediction equation; the goodness-of-fit (fitting error) is evaluated on these 50 data points. The obtained prediction equation is then used to forecast the remaining 10 observations, and the prediction error is evaluated on those 10 points.

### Proposed GM(0,N) model

1. Modeling Procedure based on combined exponential and trigonometric transformations

The dependent variable sequence is denoted as${\mathrm{X}}_{{\mathrm{1}}}^{{{\mathrm{(0)}}}}$, while the *N* − 1 independent variable sequences are represented as ${\mathrm{X}}_{{\mathrm{2}}}^{{{\mathrm{(0)}}}}{\mathrm{,~X}}_{{\mathrm{3}}}^{{{\mathrm{(0)}}}}{\text{,~ \ldots ,~X}}_{{\mathrm{N}}}^{{{\mathrm{(0)}}}}$. The dependent sequence ${\mathrm{X}}_{{\mathrm{1}}}^{{{\mathrm{(0)}}}}{\text{=\{ x}}_{{\mathrm{1}}}^{{{\mathrm{(0)}}}}\left( {\mathrm{1}} \right){\mathrm{,x}}_{{\mathrm{1}}}^{{{\mathrm{(0)}}}}\left( {\mathrm{2}} \right){\text{, \ldots ,x}}_{{\mathrm{1}}}^{{{\mathrm{(0)}}}}\left( {\mathrm{n}} \right){\mathrm{\} }}$ is preprocessed so that its values fall within the interval $\left( {{\mathrm{0,}}\frac{{\mathrm{\boldsymbol{\uppi}}}}{{\mathrm{2}}}} \right)$. The transformed sequence is expressed as ${\mathrm{Y}}_{{\mathrm{1}}}^{{{\mathrm{(0)}}}}{\text{=\{ y}}_{{\mathrm{1}}}^{{{\mathrm{(0)}}}}\left( {\mathrm{1}} \right){\mathrm{,y}}_{{\mathrm{1}}}^{{{\mathrm{(0)}}}}\left( {\mathrm{2}} \right){\text{, \ldots ,y}}_{{\mathrm{1}}}^{{{\mathrm{(0)}}}}\left( {\mathrm{n}} \right){\mathrm{\} }}$, and the transformation is defined as follows:

12 $${\mathrm{y}}_{{\mathrm{1}}}^{{{\mathrm{(0)}}}}\left( {\mathrm{k}} \right){\mathrm{=}}\frac{{{\mathrm{x}}_{{\mathrm{1}}}^{{{\mathrm{(0)}}}}\left( {\mathrm{k}} \right)}}{{\mathrm{M}}},\;k=1,{\text{ }}2,{\text{ }}...,{\text{ }}n$$

where, *M* is an unknown parameter used in the preprocessing of the original sequence, ${\mathrm{x}}_{{\mathrm{1}}}^{{{\mathrm{(0)}}}}\left( {\mathrm{k}} \right)$ represents the value before preprocessing, ${\mathrm{y}}_{{\mathrm{1}}}^{{{\mathrm{(0)}}}}\left( {\mathrm{k}} \right)$ represents the value after preprocessing. Since the transformed sequence must fall within the interval $\left( {{\mathrm{0,}}\frac{{\mathrm{\boldsymbol{\uppi}}}}{{\mathrm{2}}}} \right)$, it is required that ${\mathrm{max}}\left\{ {\frac{{{\mathrm{x}}_{{\mathrm{1}}}^{{{\mathrm{(0)}}}}\left( {\mathrm{k}} \right)}}{{\mathrm{M}}}} \right\}{\mathrm{<}}\frac{{\mathrm{\boldsymbol{\uppi}}}}{{\mathrm{2}}}$.

Based on this constraint, the minimum value of the parameter *M*, denoted as *h*, can be determined, where (*M* > *h*). The calculation method for *h* is given as follows:

13 $${\mathrm{h=}}\frac{{{\mathrm{2}} \cdot {\mathrm{max}}\left\{ {{\mathrm{x}}_{{\mathrm{1}}}^{{{\mathrm{(0)}}}}\left( {\mathrm{k}} \right)} \right\}}}{{\mathrm{\boldsymbol{\uppi}}}},\;k{\text{ }}={\mathrm{1}},{\text{ 2}},{\text{ }}...,{\text{ }}n$$

where, *h* is the minimum value of the variable parameter *M*. The sequence ${\mathrm{Y}}_{{\mathrm{1}}}^{{{\mathrm{(0)}}}}$ is further transformed to obtain a new sequence ${\mathrm{S}}_{{\mathrm{1}}}^{{{\mathrm{(0)}}}}{\mathrm{=}}\left\{ {{\mathrm{s}}_{{\mathrm{1}}}^{{{\mathrm{(0)}}}}\left( {\mathrm{1}} \right){\mathrm{,~s}}_{{\mathrm{1}}}^{{{\mathrm{(0)}}}}\left( {\mathrm{2}} \right){\text{,~ \ldots ,~s}}_{{\mathrm{1}}}^{{{\mathrm{(0)}}}}\left( {\mathrm{n}} \right)} \right\}$, which is calculated in Eq. (14):

14 $${\mathrm{s}}_{{\mathrm{1}}}^{{{\mathrm{(0)}}}}\left( {\mathrm{k}} \right)={{\mathrm{c}}^{ - {\mathrm{tan}}\left( {{\mathrm{y}}_{{\mathrm{1}}}^{{{\mathrm{(0)}}}}\left( {\mathrm{k}} \right)} \right)}},\;k{\text{ }}={\mathrm{1}},{\text{ 2}},{\text{ }}...,{\text{ }}n$$

By substituting Eq. (12) into Eq. (14), the new formula can be obtained.

15 $${\mathrm{s}}_{{\mathrm{1}}}^{{{\mathrm{(0)}}}}\left( {\mathrm{k}} \right)={{\mathrm{c}}^{ - {\mathrm{tan}}\left( {{\mathrm{x}}_{{\mathrm{1}}}^{{{\mathrm{(0)}}}}\left( {\mathrm{k}} \right)/{\mathrm{M}}} \right)}},\;k{\text{ }}={\mathrm{1}},{\text{ 2}},{\text{ }}...,{\text{ }}n$$

where, *c* denotes the undetermined base of the exponential function, with the requirement that *c* > 1, ${\mathrm{s}}_{{\mathrm{1}}}^{{{\mathrm{(0)}}}}\left( {\mathrm{k}} \right)$ is the new sequence obtained through the combined transformation of the exponential and trigonometric functions.

Through this step, the preprocessed sequence ${\mathrm{S}}_{{\mathrm{1}}}^{{{\mathrm{(0)}}}}$ is obtained. The subsequent modeling procedure follows the same steps as the GM(0,N) model, namely: performing first-order accumulation, constructing the linear model, estimating the parameters ${{\mathrm{b}}_{\mathrm{0}}}{\mathrm{,~}}{{\mathrm{b}}_{\mathrm{2}}}{\mathrm{,~}}{{\mathrm{b}}_{\mathrm{3}}}{\mathrm{,~}}...{\mathrm{,~}}{{\mathrm{b}}_{\mathrm{N}}}$ using the least squares method, and restoring the sequence to obtain ${{\hat {\mathrm{S}}}}_{{\mathrm{1}}}^{{\left( {\mathrm{0}} \right)}}$​. Since the original sequence has been preprocessed using a composite function, an additional inverse transformation is required after obtaining the prediction sequence ${{\hat {\mathrm{S}}}}_{{\mathrm{1}}}^{{\left( {\mathrm{0}} \right)}}$. By taking the natural logarithm on both sides of Eq. (15) and performing simple mathematical rearrangements, ${{\hat {\mathrm{x}}}}_{{\mathrm{1}}}^{{\left( {\mathrm{0}} \right)}}\left( {\mathrm{k}} \right)$ is obtained. The restored prediction sequence ${{\hat {\mathrm{X}}}}_{{\mathrm{1}}}^{{\left( {\mathrm{0}} \right)}}$ is then derived according to Eq. (16):

16 $${{\hat {\mathrm{x}}}}_{{\mathrm{1}}}^{{\left( {\mathrm{0}} \right)}}\left( {\mathrm{k}} \right){\mathrm{=M}} \cdot {\mathrm{arctan}}\left( { - \frac{{{\mathrm{In}}\left( {{{\hat {\mathrm{s}}}}_{{\mathrm{1}}}^{{\left( {\mathrm{0}} \right)}}\left( {\mathrm{k}} \right)} \right)}}{{{\mathrm{In}}\left( {\mathrm{c}} \right)}}} \right)$$

where, ${{\hat {\mathrm{s}}}}_{{\mathrm{1}}}^{{\left( {\mathrm{0}} \right)}}\left( {\mathrm{k}} \right)$ is the predicted value obtained by applying the GM(0,N) model to the sequence ${\mathrm{S}}_{{\mathrm{1}}}^{{{\mathrm{(0)}}}}$ as the initial sequence. ${{\hat {\mathrm{x}}}}_{{\mathrm{1}}}^{{\left( {\mathrm{0}} \right)}}\left( {\mathrm{k}} \right)$ is the value restored from ${{\hat {\mathrm{s}}}}_{{\mathrm{1}}}^{{\left( {\mathrm{0}} \right)}}\left( {\mathrm{k}} \right)$ using Eq. (16), and it represents the final prediction result of the GM(0,N) model proposed in this study based on the combined exponential–trigonometric transformation.

(2) Determination of parameters *c* and *M* Based on the PSO Algorithm

As described above, the preprocessing function of the GM(0,N) model contains two unknown parameters, *c* and *M*. The PSO algorithm is employed to search for the optimal values of these two parameters, thereby minimizing the prediction error.

**Step 1**: Initialization of parameters and Particle Swarm.

The mean relative error (MRE) of the GM(0,N) model predictions is adopted as the objective function. To search for the minimum of the objective function, it is calculated as follows:

17 $${\mathrm{e}}_{{\mathrm{r}}} {\text{ = }}\frac{{\mathrm{1}}}{{\mathrm{n}}}\sum\limits_{{{\text{k = 1}}}}^{{\mathrm{n}}} {\frac{{\left| {{{\hat{\mathrm{x}}}}_{{\mathrm{1}}}^{{\left( {\mathrm{0}} \right)}} \left( {\mathrm{k}} \right) - {\mathrm{x}}_{{\mathrm{1}}}^{{{\mathrm{(0)}}}} \left( {\mathrm{k}} \right)} \right|}}{{\left| {{\mathrm{x}}_{{\mathrm{1}}}^{{{\mathrm{(0)}}}} \left( {\mathrm{k}} \right)} \right|}}}$$

where, ${{\hat {\mathrm{x}}}}_{{\mathrm{1}}}^{{\left( {\mathrm{0}} \right)}}\left( {\mathrm{k}} \right)$ denotes the predicted value, ${\mathrm{x}}_{{\mathrm{1}}}^{{{\mathrm{(0)}}}}\left( {\mathrm{k}} \right)$ represents the actual value, ${{\mathrm{e}}_{\mathrm{r}}}$ is the MRE, and *n* is the number of elements in the sequence.

The algorithm parameters are set as follows. Piotrowski et al.^44^ indicated that the optimal particle population size ranges from 20 to 50. In this study, experimental results show that a population size of 40 provides better search performance and faster convergence. Tarekegn et al.^45^ recommended setting the inertia weight ω to 0.7298, and the learning factors, the cognitive coefficient ${{\mathrm{c}}_{\mathrm{1}}}$​ and the social coefficient ${{\mathrm{c}}_{\mathrm{2}}}$​, to 2. The maximum number of iterations *T* is set to 50, and experimental results confirmed that this value ensures convergence for all datasets used in this study. The hyperparameter settings and parameter selection methods are based on Piotrowski et al.^44^, and no additional limits are imposed on particle velocity.

To optimize the two unknown parameters using the PSO algorithm, the feasible ranges for the parameters are defined. Based on the previous analysis, *M* > *h*, and multiple experiments show that the optimal values of *M* generally fall within the range 10*h*. Therefore, the final range for *M* is set as (*h*,10*h*). For ccc, since *c* > 1 and experiments indicate that values greater than 10 have little impact on prediction accuracy, the range is empirically set to (1,10).

The particles are initialized by generating 40 two-dimensional vectors to represent the initial positions and velocities. Each particle’s position is denoted as ${{\mathrm{x}}_{\mathrm{i}}}{\mathrm{~=~}}\left( {{{\mathrm{x}}_{{\mathrm{i1}}}}{\mathrm{,~}}{{\mathrm{x}}_{{\mathrm{i2}}}}{\mathrm{,~}}{{\mathrm{x}}_{{\mathrm{i3}}}}{\mathrm{,~}} \ldots {\mathrm{,~}}{{\mathrm{x}}_{{\mathrm{iD}}}}} \right)$, and its velocity as ${{\mathrm{v}}_{\mathrm{i}}}{\mathrm{~=~}}\left( {{{\mathrm{v}}_{{\mathrm{i1}}}}{\mathrm{,~}}{{\mathrm{v}}_{{\mathrm{i2}}}}{\mathrm{,~}}{{\mathrm{v}}_{{\mathrm{i3}}}}{\mathrm{,~}} \ldots {\mathrm{,~}}{{\mathrm{v}}_{{\mathrm{iD}}}}} \right)$, where the initial positions of the particles must lie within the predefined parameter ranges.

**Step 2**: Initialization of Individual and Global Best.

The initial fitness is calculated as follows: for each particle *i*, after every iteration, a position ${{\mathrm{x}}_{\mathrm{i}}}$ is generated. Substituting ${{\mathrm{x}}_{\mathrm{i}}}$​ into the fitness function yields a corresponding fitness value.

Individual Best (pbest): For particle iii, the position among all previously visited positions that yields the minimum fitness is selected as the individual best vector ${{\mathrm{p}}_{\mathrm{i}}}$, ${{\mathrm{p}}_{{\mathrm{i~}}}}{\mathrm{=~}}\left( {{{\mathrm{p}}_{{\mathrm{i1}}}}{\mathrm{,~}}{{\mathrm{p}}_{{\mathrm{i2}}}}{\mathrm{,~}} \ldots {\mathrm{,~}}{{\mathrm{p}}_{{\mathrm{iD}}}}} \right){\mathrm{,~i=1,~}} \cdots {\mathrm{,~N}}$.

Global Best (gbest): Among all individual best vectors, the one with the minimum fitness is selected as the global best vector for the swarm ${{\mathrm{p}}_{\mathrm{g}}}$, ${{\mathrm{p}}_{{\mathrm{g~}}}}{\mathrm{=~}}\left( {{{\mathrm{p}}_{{\mathrm{g1}}}}{\mathrm{,~}}{{\mathrm{p}}_{{\mathrm{g2}}}}{\mathrm{,~}} \ldots {\mathrm{,~}}{{\mathrm{p}}_{{\mathrm{gD}}}}} \right)$, where ${{\mathrm{p}}_{\mathrm{g}}}$ is unique.

**Step 3**: Iterative Optimization.

Velocity Update: Each particle updates its velocity based on its individual best (${{\mathrm{p}}_{\mathrm{i}}}$ ) and the global best (${{\mathrm{p}}_{\mathrm{g}}}$​) according to Eq. (18):

18 $${\mathrm{v}}_{{{\mathrm{ij}}}}^{{{\mathrm{k+1}}}}{\mathrm{~=~\boldsymbol{\upomega}v}}_{{{\mathrm{ij}}}}^{{{\mathrm{k~}}}}{\mathrm{+~}}{{\mathrm{c}}_{\mathrm{1}}}{{\mathrm{r}}_{\mathrm{1}}}\left( {{\mathrm{p}}_{{{\mathrm{ij}}}}^{{\mathrm{k}}} - {\mathrm{x}}_{{{\mathrm{ij}}}}^{{\mathrm{k}}}} \right)+{{\mathrm{c}}_{\mathrm{2}}}{{\mathrm{r}}_{\mathrm{2}}}\left( {{\mathrm{p}}_{{{\mathrm{gj}}}}^{{\mathrm{k}}} - {\mathrm{x}}_{{{\mathrm{ij}}}}^{{\mathrm{k}}}} \right)$$

where, *k* denotes the current iteration; ${\mathrm{P}}_{{{\mathrm{ij}}}}^{{\mathrm{k}}}$ ​ is the individual best value of the *j*-th variable for particle *i* at iteration *k*; ${\mathrm{P}}_{{{\mathrm{gj}}}}^{{\mathrm{k}}}$ is the global best value of the *j*-th variable at iteration *k*; ${\mathrm{x}}_{{{\mathrm{ij}}}}^{{\mathrm{k}}}$​ is the current value of the *j*-th variable for particle *i* at iteration *k*; ${\mathrm{v}}_{{{\mathrm{ij}}}}^{{\mathrm{k}}}$​ is the velocity of the *j*-th variable for particle *i* at iteration *k*; and ${\mathrm{v}}_{{{\mathrm{ij}}}}^{{{\mathrm{k+1}}}}$ is the velocity of the *j*-th variable for particle *i* at iteration *k* + 1.

Position Update: Each particle updates its position according to its new velocity, as expressed in Eq. (19):

19 $${\mathrm{x}}_{{{\mathrm{ij}}}}^{{{\mathrm{k+1}}}}={\mathrm{x}}_{{{\mathrm{ij}}}}^{{\mathrm{k}}}+{\mathrm{v}}_{{{\mathrm{ij}}}}^{{{\mathrm{k+1}}}}$$

Current Fitness Calculation: After updating, each particle’s position ${{\mathrm{x}}_{\mathrm{i}}}$ is substituted into the objective function to obtain its fitness value. To prevent particles from moving outside the predefined search space during iteration, each updated variable is checked against its bounds. Suppose the *j*-th variable has a search range [*h*,*H*]. If ${\mathrm{x}}_{{{\mathrm{ij}}}}^{{{\mathrm{k+1}}}}{\mathrm{>H}}$, then ${\mathrm{x}}_{{{\mathrm{ij}}}}^{{{\mathrm{k+1}}}}{\mathrm{=H}}$; ${\mathrm{x}}_{{{\mathrm{ij}}}}^{{{\mathrm{k+1}}}}{\mathrm{<h}}$, then ${\mathrm{x}}_{{{\mathrm{ij}}}}^{{{\mathrm{k+1}}}}{\mathrm{=h}}$. This procedure ensures that particles remain within valid ranges, allowing effective search results.

Update of Individual Best (pbest): If the current fitness of a particle is better than its historical best, the particle’s individual best ${{\mathrm{p}}_{\mathrm{i}}}$ is updated, and the corresponding fitness is recorded.

Update of Global Best (gbest): By traversing all individual bests ${{\mathrm{p}}_{\mathrm{i}}}$, if a better fitness is found, the global best ${{\mathrm{p}}_{\mathrm{g}}}$ is updated along with the corresponding fitness value.

**Step 4**: Termination Condition.

The algorithm terminates when the number of iterations reaches the preset maximum of 50. The global best position ${{\mathrm{p}}_{\mathrm{g}}}$ and its corresponding fitness value are then output, representing the approximate optimal solution to the problem.

## Data collection

**Fig. 4:**
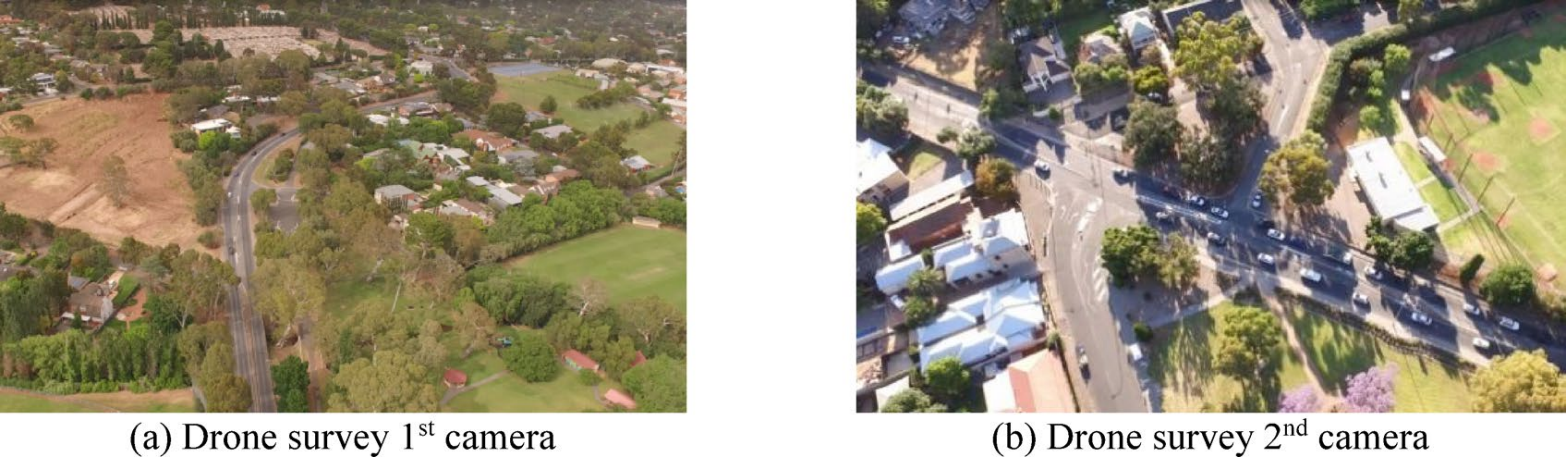
Old Belair Road metered roundabout.

The variables influencing queue length at metered roundabouts were defined based on An’s model. Among them, arrival flow, circulating flow, and green and red times vary in real time according to traffic conditions. To obtain these data, a field survey was conducted on May 19, 2023. Arrival and circulating traffic volumes were extracted from SCATS VS data, while real-time signal timings were obtained from SCATS SM data. Figure 4 illustrates the Old Belair Road roundabout where the metering system was applied during the morning peak period. Using drone observations, queue lengths at each approach were recorded for one-hour between 7:35 and 8:35 a.m by two drones. During the survey period, buses accounted for 7% and trucks for 5% of the total traffic volume, and these were converted into Passenger Car Unit (PCU) for analysis.

**Fig. 5:**
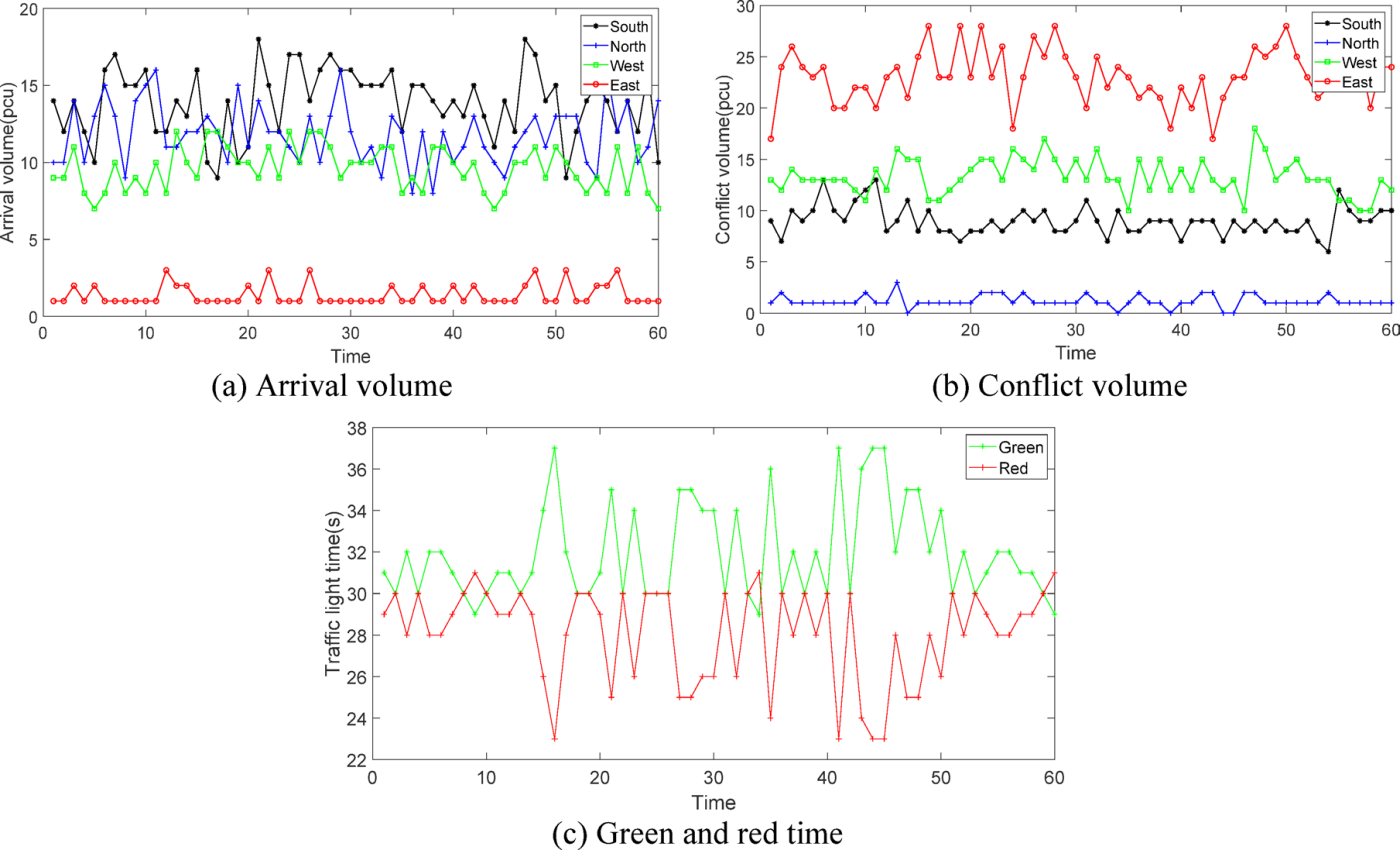
Collected data.

Figure 5 illustrates the arrival volume, conflict volume, and signal green and red durations obtained from SCAT at one-minute intervals. The observed queue lengths for each approach are presented in Figs. 7 and 8. Among the approaches, the south approach recorded the highest arrival volume, ranging from a maximum of 18 vehicles to a minimum of 9 vehicles per minute, while the east approach exhibited the lowest, with only 1–3 vehicles observed (Fig. 5a). Regarding the conflict volume, the east approach showed the highest value, reaching up to 28 vehicles, followed by the west, south, and north approaches. As shown in Fig. 5c, signal durations varied every minute, with green times ranging between 29 and 37 s and red times between 23 and 31 s.

## Result and discussion

**Fig. 6:**
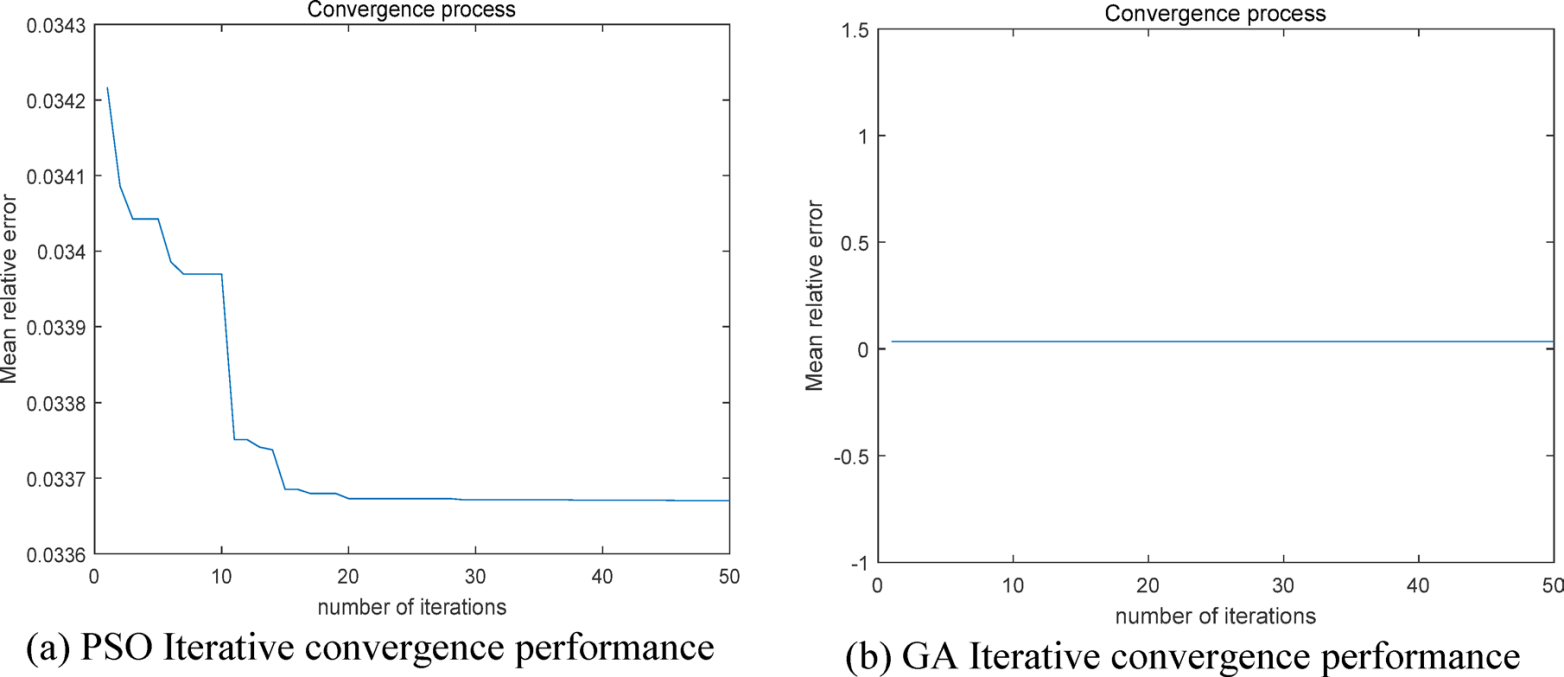
Algorithm convergence plots.

Using the west approach data of the roundabout as an example, the optimized GM(0,N) model is applied for prediction. The PSO algorithm and the GA are respectively used to search for the optimal values of the unknown parameters, and the resulting convergence plots are shown in Fig. 6.

To compare the performance of the two algorithms, three evaluation criteria were considered: the number of iterations required to reach the preset accuracy, the optimal solution obtained upon algorithm convergence, and the algorithm runtime. Each algorithm was executed five times, and the average results were reported, as summarized in Table 2. The preset accuracy was set to 0.037.

**Table 2:** Comparison of evaluation metrics for the two models.

	The number of iterations to achieve the preset accuracy	Optimal solution of algorithm iteration convergence	Algorithm running time (s)
PSO	14	0.03367	25
GA	–	0.04110	10

According to the comparative results shown in Table 2, the genetic algorithm (GA) did not reach the predefined accuracy level, whereas the particle swarm optimization (PSO) algorithm achieved a substantially better converged optimal solution. Although GA exhibited shorter computational time, this result does not indicate superior global search performance. Rather, GA is prone to premature convergence due to entrapment in local optima during the iterative search process, which results in early termination and failure to satisfy the predefined accuracy criterion. Therefore, this study adopts the PSO algorithm to determine the values of the unknown parameters. The queue lengths at the four approaches of the roundabout are predicted using An’s model, the GM(1,1) model, the GM(1,N) model, the GM(0,N) model, and the optimized GM(0,N) model. The optimized GM(0,N) model employs the PSO algorithm to determine the values of the parameters *c* and *M* for each approach, as summarized in Table 3.

**Table 3:** The values of parameters *c* and *M*.

Parameter	South	North	West	East
*c*	10	1.0011	10	9.7584
*M*	141.0533	452.8866	195.6481	31.7785

**Fig. 7:**
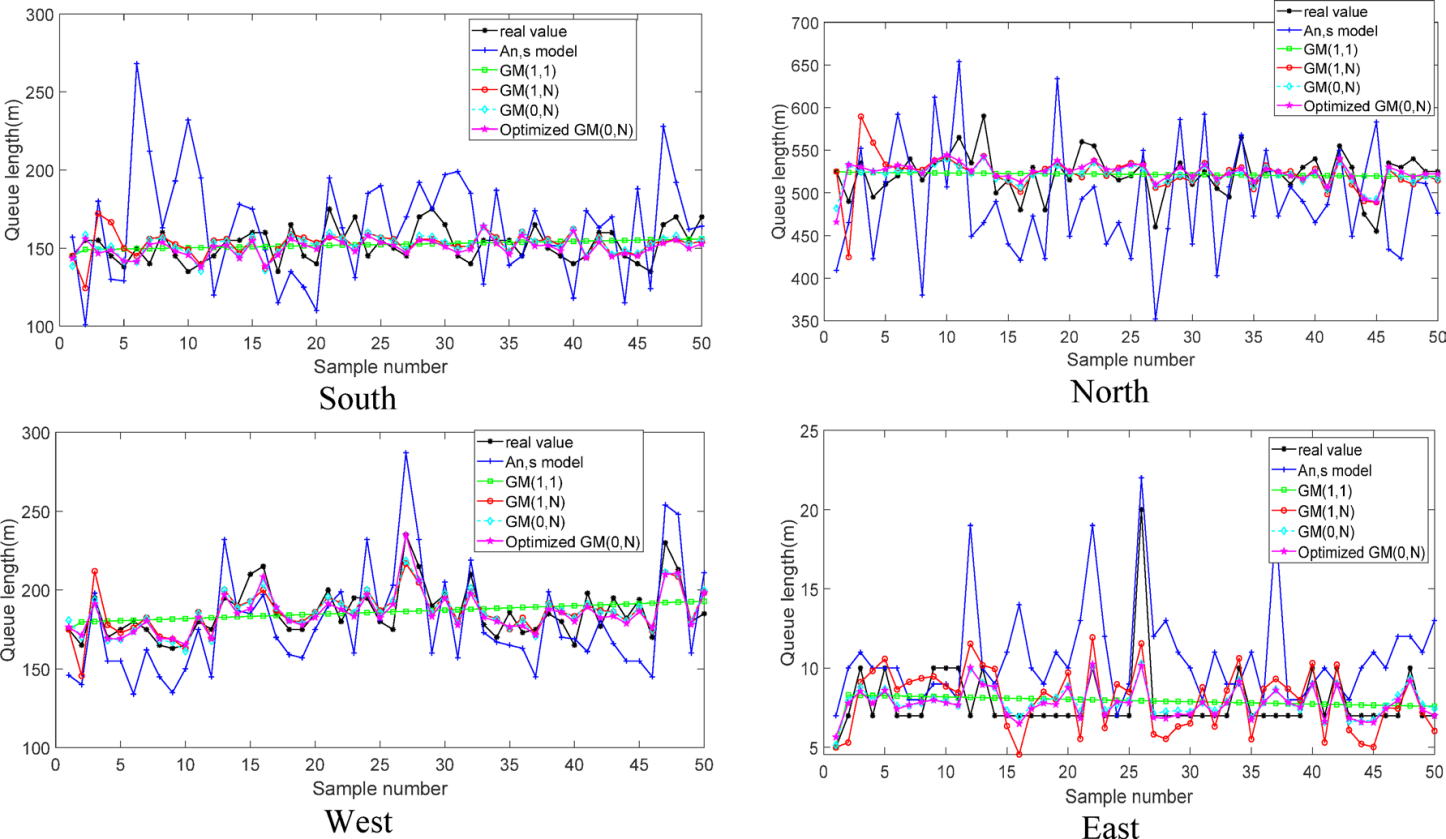
Fitting results of the training samples.

**Fig. 8:**
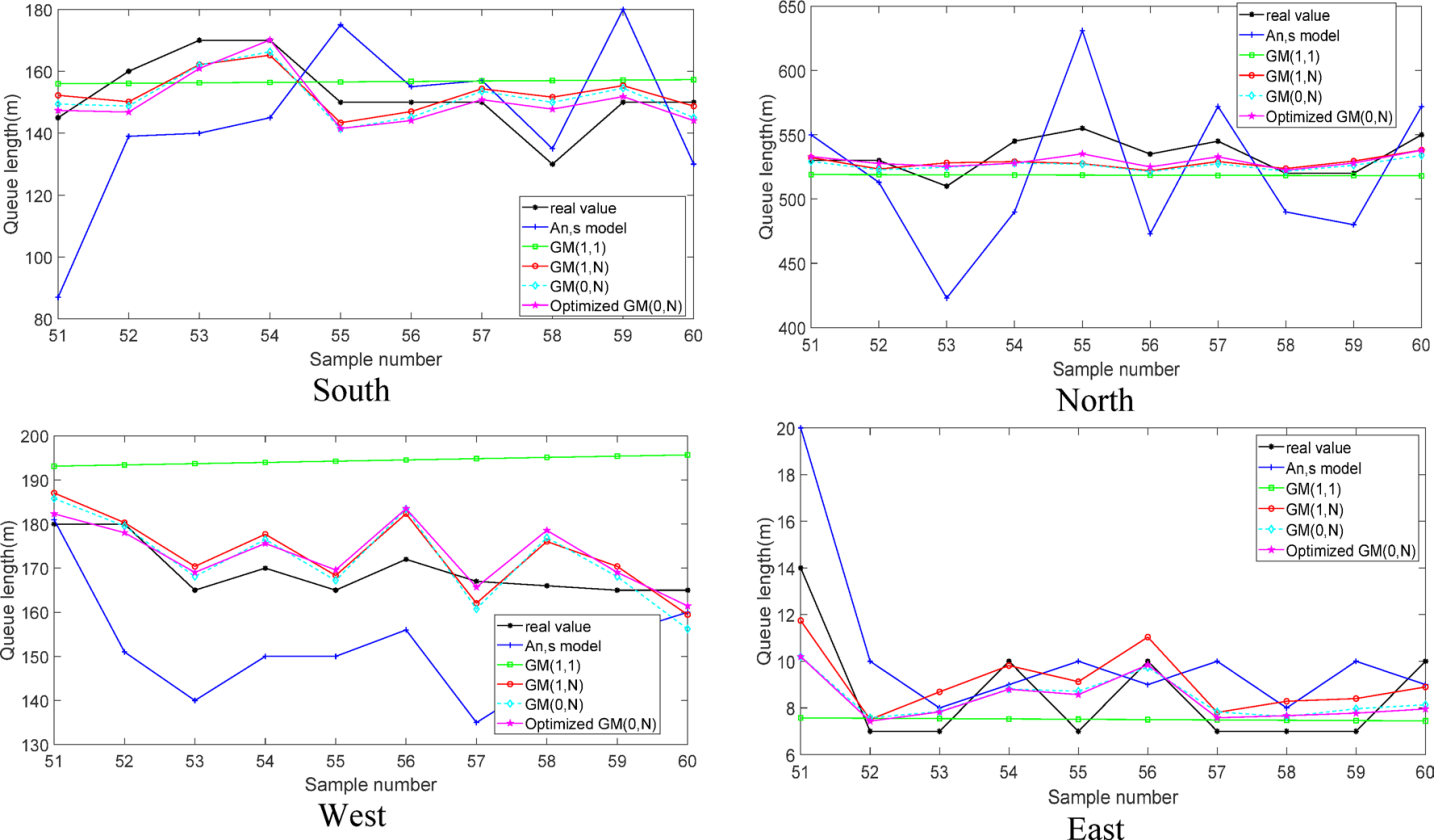
Prediction results of the test samples.

By substituting these values into the optimized GM(0,N) model, the corresponding fitting and prediction results are obtained. The fitting and prediction results obtained by the five methods are illustrated in Figs. 7 and 8.

An’s model exhibits large fluctuations in both fitting and prediction results, and none of the approaches achieve satisfactory performance. The traditional GM(1,1) model, which does not account for the influence of multiple factors on intersection queue length, can only approximate the queue length using a monotonic increasing or decreasing function based on the overall trend of the data. However, the queue lengths at the various approaches fluctuate over time, meaning that this method fails to capture the dynamic variations in the data. In particular, for the west samples, the GM(1,1) prediction trend is completely opposite to the actual trend.

In contrast, the GM(1,N) model, the GM(0,N) model, and the optimized GM(0,N) model consider multiple influencing factors, resulting in substantial improvements in both fitting and prediction accuracy compared to An’s model and the GM(1,1) model. The fitting and prediction results of these three methods can effectively reflect the variation trends of the original data and are generally satisfactory.

**Fig. 9:**
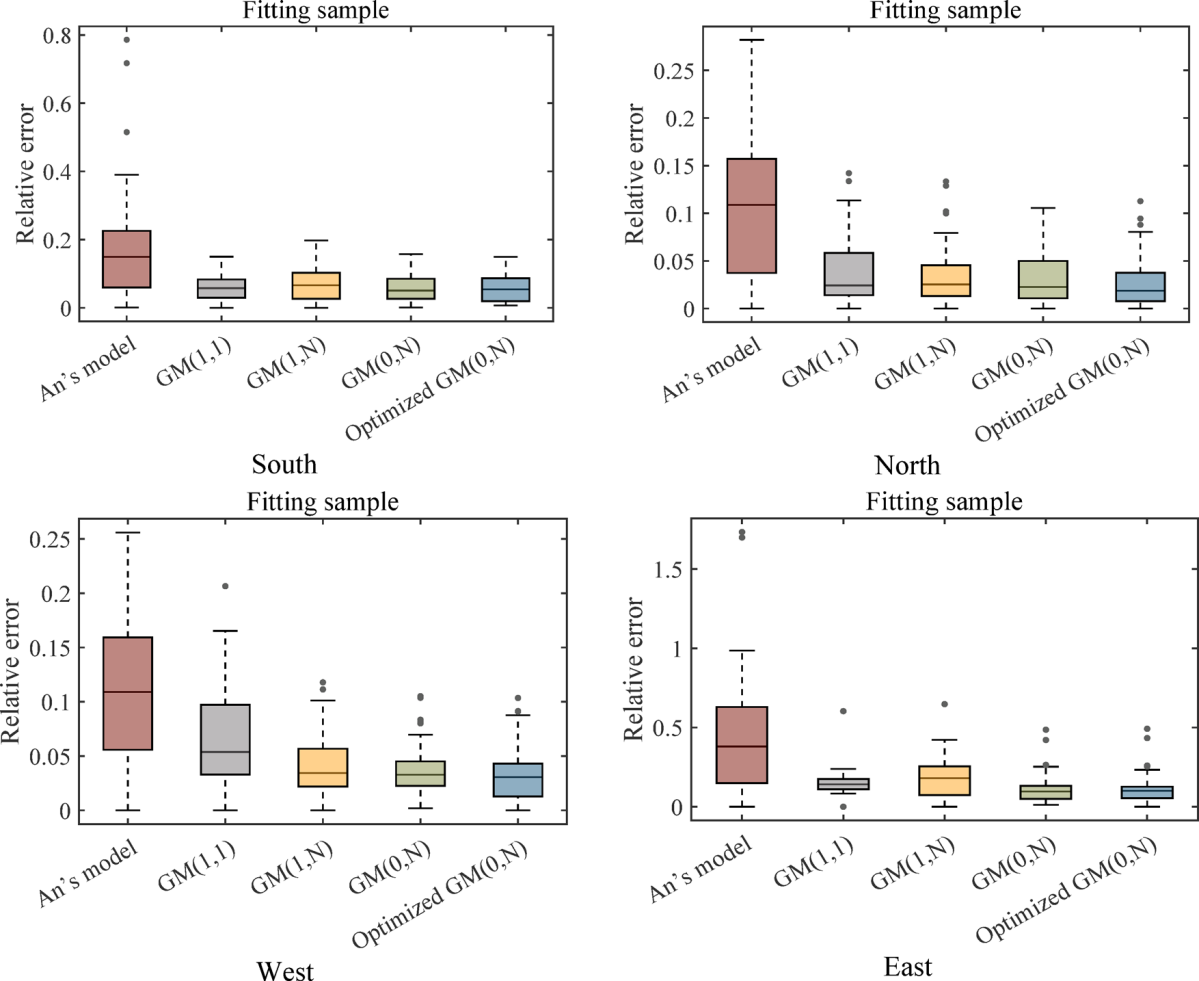
Box plots of fitting errors for different methods across the four Approaches.

To further compare the fitting and prediction accuracy among these three methods, direct visual analysis is challenging due to the large number of sample points and may introduce bias. Therefore, the deviation of each sample point from the true value is calculated, and the relative error is used to quantify the accuracy at each point. The calculation method can be expressed in Eq. (20).

20 $${\mathrm{e}}\left( {\mathrm{k}} \right)=\frac{{\left| {{{{{\hat {\mathrm{x}}}}}^{{\mathrm{(0)}}}}\left( {\mathrm{k}} \right) - {{\mathrm{x}}^{{\mathrm{(0)}}}}\left( {\mathrm{k}} \right)} \right|}}{{{{\mathrm{x}}^{{\mathrm{(0)}}}}\left( {\mathrm{k}} \right)}}$$

where, ${\mathrm{e}}\left( {\mathrm{k}} \right)$ is the relative error, ${{{\hat {\mathrm{x}}}}^{{\mathrm{(0)}}}}\left( {\mathrm{k}} \right)$ is the predicted value, ${{\mathrm{x}}^{{\mathrm{(0)}}}}\left( {\mathrm{k}} \right)$ is the actual value, and *k* represents the sample index, *k =* 1, 2, …, n. To better compare the fitting and prediction accuracy of the different methods, the relative errors were calculated and visualized using box plots. The results are shown in Fig. 9.

For the training samples, the Kruskal–Wallis test in SPSS was used to examine whether there are significant differences in the relative errors of different prediction methods across the various approaches of the roundabout. The Kruskal–Wallis test statistics for the four approaches, as calculated by the software, are shown in Table 4.

**Table 4:** Kruskal–Wallis test statistics for the four approaches.

Parameter	Approach			
	South	North	West	East
Total sample size N	250	250	250	250
Test Statistic H	34.549	53.868	61.883	61.029
Degrees of Freedom	4	4	4	4
Asymptotic Significance P (Two-Sided Test)	0.000	0.000	0.000	0.000

*N* represents the total number of samples involved. Each direction contains 50 training samples, and five prediction methods are compared, resulting in a total sample size of 250. The test statistic is the numerical value used to assess the differences among group distributions, corresponding to the H-value in the Kruskal–Wallis test. A larger value indicates stronger evidence of differences among the groups. The degrees of freedom represent the amount of “independent information” available for the statistical test. The asymptotic significance refers to the two-sided *p* value. When this value is less than 0.05, it indicates a significant difference in the distributions among the groups; when it is greater than 0.05, no significant difference is indicated.

From the results, it can be concluded that the prediction errors across the five methods differ significantly for all four approaches of the roundabout. Because at least one pair of methods shows a significant difference, a detailed pairwise comparison is necessary to identify which methods differ from one another. The SPSS-generated table for this pairwise analysis is presented in Table 5, where the five methods are labeled as 1, 2, 3, 4, and 5.

**Table 5:** Adjusted significance values for pairwise comparisons of the five methods.

Method	South	North	West	East
5-4	1	1	1	1
5 -3	1	1	1	0.005
5 -2	1	1	0.002	0.018
5 -1	0.000	0.000	0.000	0.000
4 -3	1	1	1	0.013
4 -2	1	1	0.014	0.044
4 -1	0.000	0.000	0.000	0.000
3 -2	1	1	0.66	1
3 -1	0.001	0.000	0.000	0.010
2 -1	0.000	0.000	0.033	0.003

Note: Method 1 (An’s model); Method 2 (GM(1,1)); Method 3 (GM(1,N)); Method 4 (GM(0,N)); Method 5 (Optimized GM(0,N)).

With respect to the south approach, all pairwise comparisons among the methods except An’s model show significance values of 1, indicating no significant differences among these four methods. In contrast, all comparisons between An’s model and the other methods yield significance values below 0.05, suggesting that An’s model differs significantly from the remaining methods. When viewed alongside the boxplot patterns, it can be concluded that An’s model exhibits larger fitting errors, while the other four methods show smaller and relatively similar fitting errors. For the north approach, the significance values are almost identical to those of the South approach, leading to the same conclusion that An’s model has larger fitting errors and the other four models have smaller and comparable errors. In terms of the west approach, the same general conclusion applies and An’s model again shows noticeably larger fitting errors. In addition, the table shows that both the Optimized GM(0,N) model and the GM(0,N) model exhibit significant differences when compared with the GM(1,1) model. Combined with the boxplot analysis, this indicates that the Optimized GM(0,N) model and the GM(0,N) model perform substantially better than the GM(1,1) model. The GM(1,N) model does not show significant differences from either group, meaning from both the Optimized GM(0,N) model and GM(0,N) model on one side and from the GM(1,1) model on the other side. When interpreted with the boxplots, its fitting errors fall between these two groups. For the east approach, the Kruskal–Wallis test results indicate no statistically significant differences among the validation results of the models, with overall fitting errors being similar and generally large. According to the boxplot distributions, the optimized GM(0,N) model and the GM(0,N) model still exhibit relatively higher fitting accuracy, followed by the GM(1,N) and GM(1,1) models, whereas An’s model shows the lowest fitting accuracy. This phenomenon can be mainly attributed to the relatively low traffic demand in the east approach, the distinct spatiotemporal distribution characteristics of conflicting traffic flows compared with other approaches, and the presence of measurement uncertainty in the observed data. These factors collectively reduce the discriminative power among models, resulting in similar and generally high prediction errors for east approach traffic demand.

Analyzing the box plots of fitting errors for the four approaches and excluding outliers, it is evident that, for all approaches, An’s model exhibits a wider range of relative errors compared to the other models. Specifically, key parameters such as the range between the whiskers, the interquartile range, the upper whisker, upper quartile, median, and lower quartile are all larger than those of the other models, indicating that An’s model has the poorest fitting performance, consistent with the conclusions drawn above.

By combining the boxplot results with the significance analysis from the Kruskal–Wallis test, the final fitting accuracy ranking for the four approaches is as follows:

- South: An’s model< (GM(1,N), GM(1,1),GM(0,N), Optimized GM(0,N)).
- North: An’s model< (GM(1,N), GM(1,1),GM(0,N), Optimized GM(0,N)).
- West: An’s model < GM(1,1) < GM(1,N)< (GM(0,N), Optimized GM(0,N).
- East: An’s model< (GM(1,N), GM(1,1))< (GM(0,N), Optimized GM(0,N)).

In cases where the key box plot parameters are similar, the methods are considered to have comparable relative errors and are grouped together in parentheses in the ranking.

**Fig. 10:**
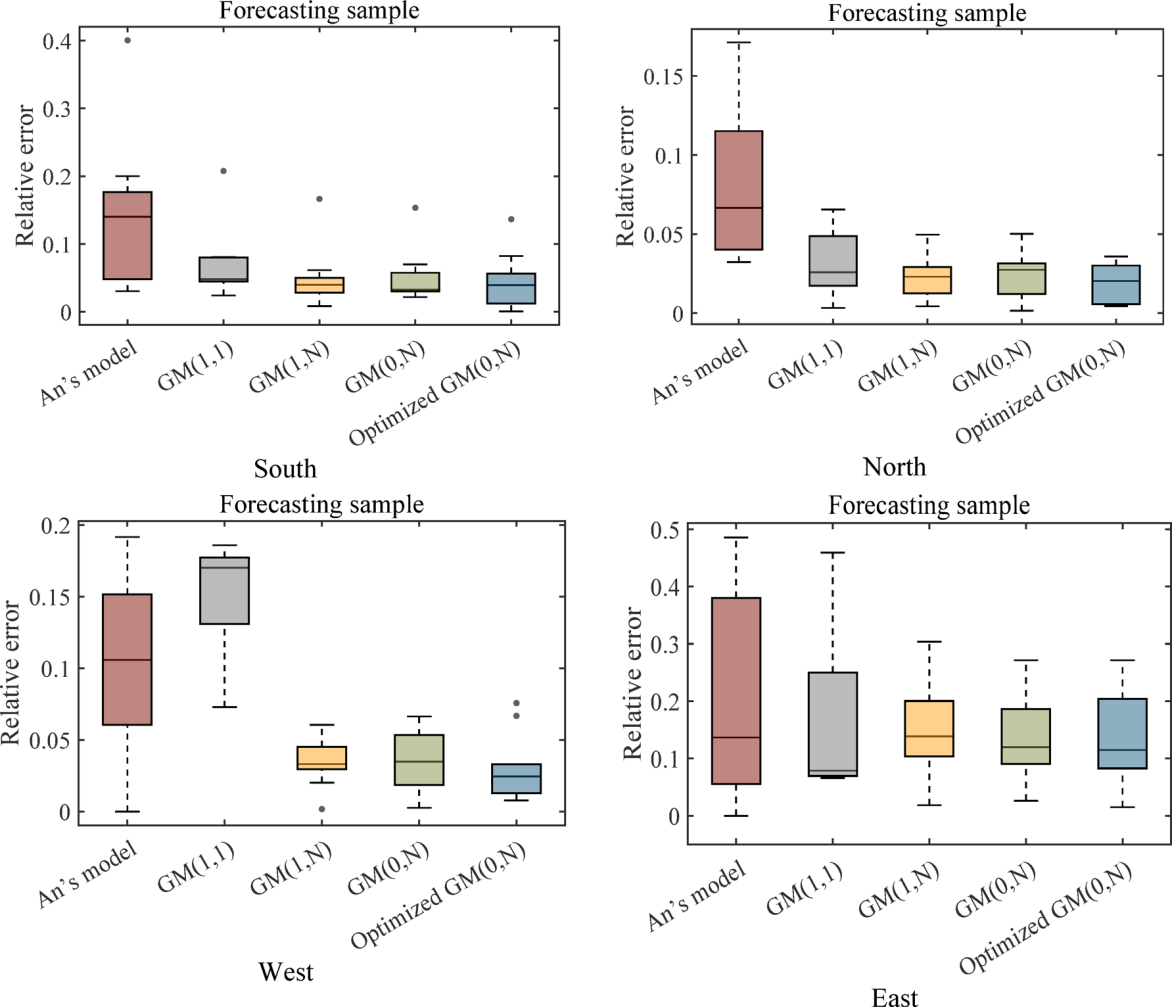
Box plots of prediction errors for different methods across the four approaches.

Based on the fitting accuracy rankings across all four approaches, the optimized GM(0,N) model consistently achieves the highest tier of accuracy, demonstrating the effectiveness of the optimization approach proposed in this study. Meanwhile, the GM(1,1) model achieves higher fitting accuracy than GM(1,N) for the south and east approaches, indicating that although GM(1,1) cannot fully capture data fluctuations, it can still approximate the true values sufficiently to achieve reasonable fitting accuracy (Fig. 10).

For the validation samples, the Kruskal–Wallis test was conducted in SPSS to examine the significant differences in relative errors among the various prediction methods for each approach of the roundabout. The Kruskal–Wallis test statistics for the four approaches, as computed by the software, are presented in Table 6.

**Table 6:** Kruskal–Wallis test statistics for the four approaches.

Parameter	Approach			
	South	North	West	East
Total sample size N	50	50	50	50
Test Statistic H	11.110	19.128	26.906	3.681
Degrees of Freedom	4	4	4	4
Asymptotic Significance P (Two-Sided Test)	0.025	0.001	0.000	0.451

Based on the significance values of the Kruskal–Wallis test, significant differences are found among the methods for the south, north, and west approaches. In contrast, no significant differences are observed among the methods for the East approach. Combined with the boxplot analysis, this indicates that the prediction errors of the five methods for the east approach are relatively similar and generally large. To further examine the significance patterns for the south, north, and west approaches, the adjusted significance values between each pair of methods were obtained using SPSS, as shown in Table 7.

**Table 7:** Adjusted significance values for pairwise comparisons for the three approaches.

Method	South	North	West
5 -4	1	1	1
5 -3	1	1	1
5 -2	1	1	0.000
5 -1	0.63	0.002	0.055
4 -3	1	1	1
4 -2	1	1	0.001
4 -1	0.130	0.005	0.154
3 -2	1	1	0.002
3 -1	0.091	0.006	0.261
2 -1	1	0.060	1

Note: Method 1 (An’s model); Method 2 (GM(1,1)); Method 3 (GM(1,N)); Method 4 (GM(0,N)); Method 5 (Optimized GM(0,N)).

In terms of the south approach, although the unadjusted significance values indicate differences among the methods, most statistical analyses rely on the adjusted significance values. According to the table, all adjusted significance values exceed 0.05, suggesting no significant differences after adjustment. However, the significance values for the comparisons between GM(1,N), GM(0,N), and Optimized GM(0,N) against An’s model are close to the boundary value of 0.05. Combined with the boxplot analysis, this indicates that GM(1,N), GM(0,N), and Optimized GM(0,N) perform better than An’s model. The GM(1,1) model does not show significant differences from the other methods, placing its performance between the two groups identified above. For the North approach, significant differences are observed between An’s model and the GM(1,N), GM(0,N), and Optimized GM(0,N) models, indicating that An’s model has the lowest prediction accuracy. Although the GM(1,1) model does not show a significant difference from An’s model, its significance value is close to the critical threshold of 0.05. Combined with the boxplot analysis, it can be concluded that the GM(1,1) model performs better than An’s model, but remains inferior to the other three methods. For the West approach, no significant differences are found among the GM(1,N), GM(0,N), and Optimized GM(0,N) models, and similarly, no significant differences are observed between the GM(1,1) model and An’s model. Combined with the boxplot analysis, it can be concluded that the GM(1,N), GM(0,N), and Optimized GM(0,N) models outperform the GM(1,1) model and An’s model. With respect to the East approach, since no significant differences are observed among the methods, the conclusion is drawn directly from the boxplot analysis. An’s model shows the lowest prediction accuracy, followed by the GM(1,1) model, while the remaining three methods exhibit the highest and relatively similar prediction accuracy.

The prediction accuracy rankings are as follows:

- South: An’s model < GM(1,1)< (GM(1,N), GM(0,N)), Optimized GM(0,N))
- North: An’s model < GM(1,1)< (GM(1,N), GM(0,N), Optimized GM(0,N)).
- West: (GM(1,1),An’s model)< (GM(1,N), GM(0,N)), Optimized GM(0,N))
- East: An’s model < GM(1,1)< (GM(1,N), GM(0,N)), Optimized GM(0,N))

From the box plots of prediction results, it is clear that the optimized GM(0,N) model consistently achieves the highest tier of prediction accuracy across all approaches, which is consistent with the analysis of the fitting results. An’s model produces the poorest prediction results for the south, north, and east approaches, while the GM(1,1) model exhibits the lowest accuracy for the west approach, confirming the conclusions drawn from the line chart analysis.

Since box plots only reflect the range of relative errors and some key statistical points for all samples, they allow only a preliminary assessment of the methods’ performance. When parameters are similar, direct comparisons of method accuracy are not reliable. This study employs RMSE, MAE, and MRE as joint evaluation criteria, leveraging their complementary properties in terms of physical interpretability, dimensional characteristics, and sensitivity to prediction errors. RMSE and MAE preserve the physical units of the original observations and thus serve as absolute error measures that intuitively quantify the magnitude of prediction deviations. MAE provides a robust estimate of the average absolute deviation and is less affected by outliers, whereas RMSE assigns greater weight to large errors, enabling effective identification of extreme prediction deviations and reflecting the model’s performance under abnormal operating conditions or outlier scenarios. In contrast, MRE is a dimensionless relative error metric that eliminates biases associated with physical units and magnitude scales, facilitating accuracy comparisons across variables and scales. Consequently, RMSE and MAE ensure engineering interpretability and physical relevance, while MRE ensures generalizability and comparability. Their combined application provides a multidimensional, unbiased, and rigorous evaluation framework for prediction accuracy.

**Fig. 11:**
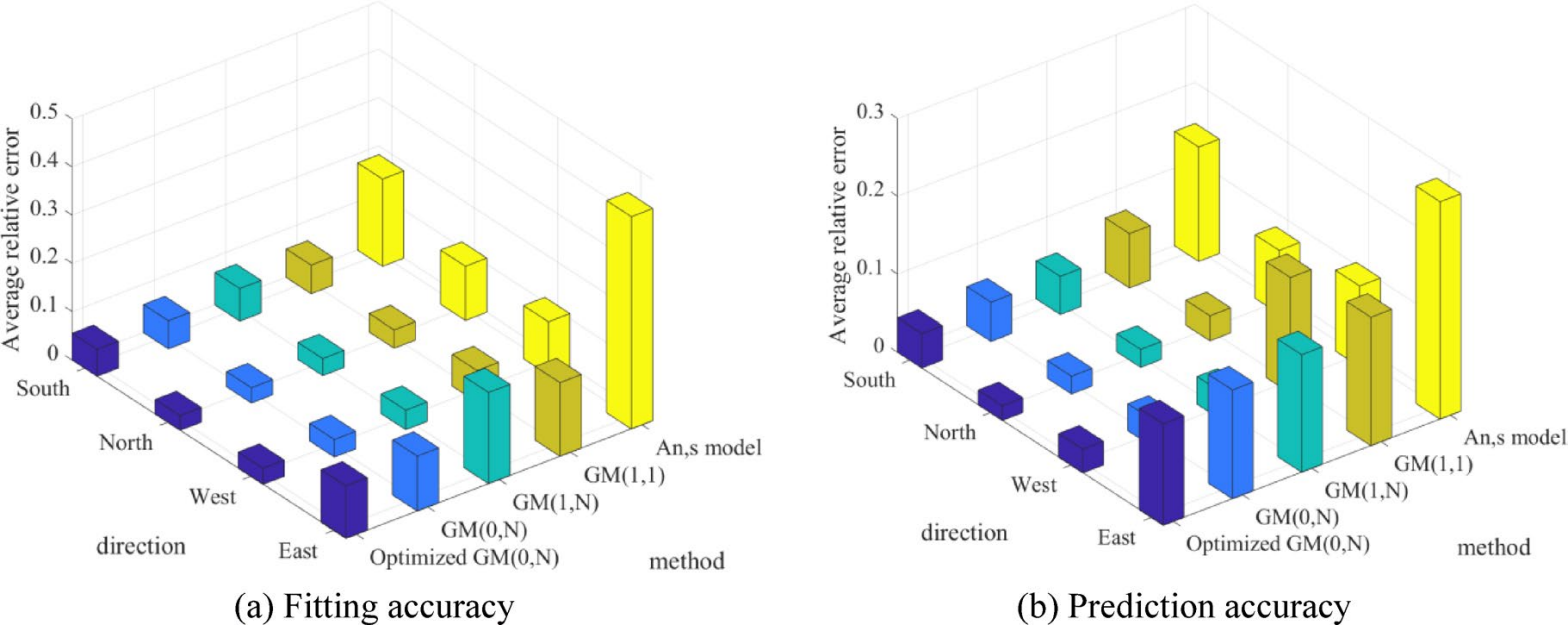
Comparison between four approaches.

The MRE across all fitting and prediction samples was calculated for each method to obtain a quantitative measure of average accuracy. The main results are shown in Fig. 11.

The MRE of the samples corresponding to different methods and directions can be obtained. For the fitting samples, the optimized GM(0,N) model achieves the highest fitting accuracy, with MREs of 0.0558, 0.0306, 0.0337, and 0.1093 for the south, north, west, and east approaches, respectively. The next best model is the GM(0,N) model, with corresponding MREs of 0.0577, 0.0317, 0.0364, and 0.1143.

Similarly, for the prediction samples, the optimized GM(0,N) model also achieves the highest prediction accuracy, with MREs of 0.0443, 0.0190, 0.0306, and 0.1305 for the south, north, west, and east approaches, respectively. The GM(0,N) model generally performs slightly lower than GM(1,N) for the south approach (0.0500 vs. 0.0490), but outperforms GM(1,N) for the other approaches, with MREs of 0.0500, 0.0228, 0.0347, and 0.1399 for the four approaches.

**Fig. 12:**
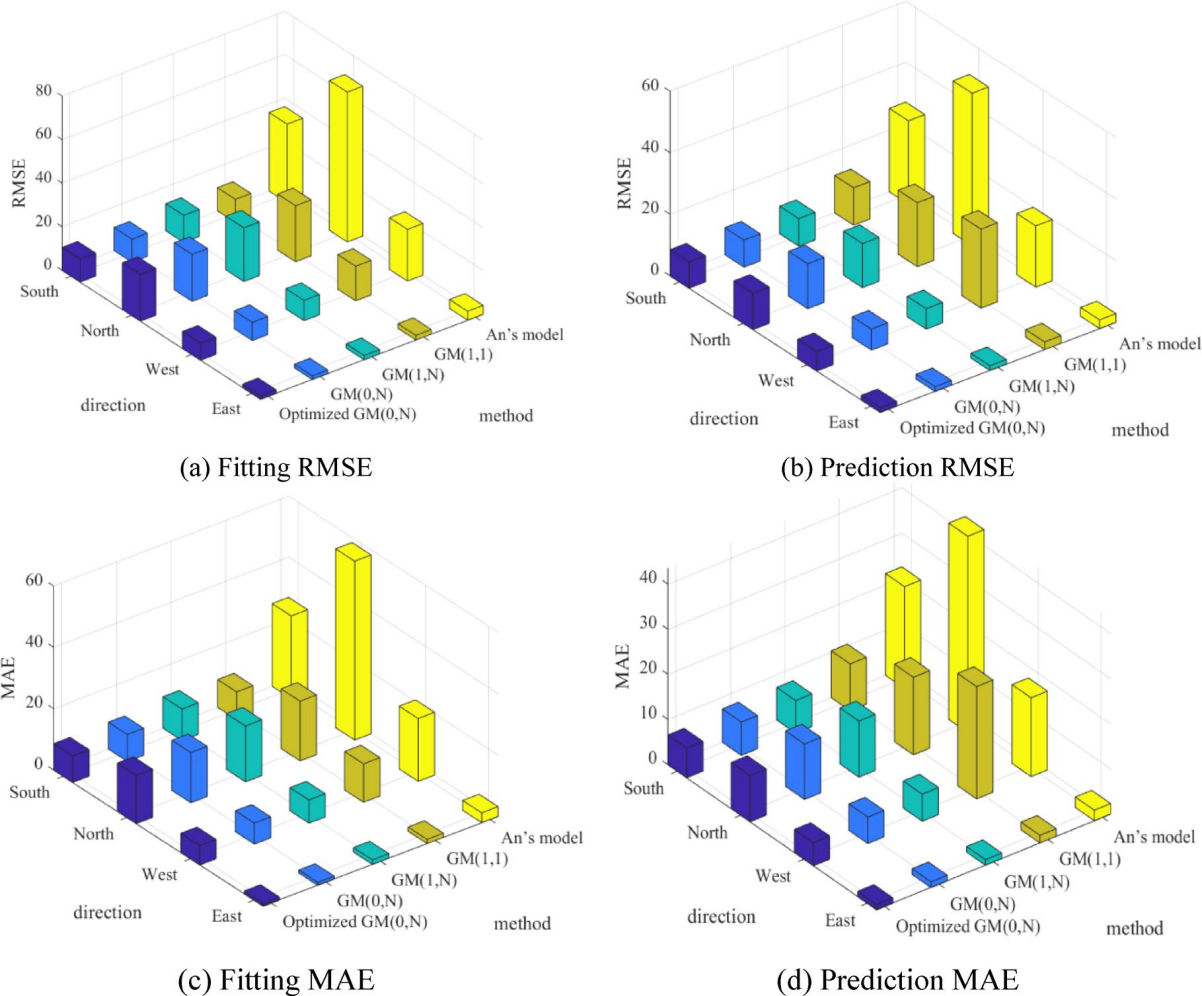
Comparison of RMSE and MAE.

After further comparing the MRE, the RMSE and MAE indicators were computed for different approaches, different methods, and different samples of the roundabout. The calculation formulas for the RMSE and MAE are shown below.

21 $$RMSE=\sqrt {\frac{1}{n}\mathop \sum \limits_{{i=1}}^{n} {{\left( {{y_i} - {{\hat {y}}_i}} \right)}^2}}$$

22 $$MAE=\frac{1}{n}\mathop \sum \limits_{{i=1}}^{n} \left| {{y_i} - {{\hat {y}}_i}} \right|$$

where, *n* is the number of samples, ${{\mathrm{y}}_{\mathrm{i}}}$​ is the true value of the *i*-th sample, and ${{\hat{\mathrm{y}}}}_{{\mathrm{i}}}$ is the predicted value of the *i*-th sample. By substituting the results from the fitting and validation samples into the equations, the RMSE and MAE indicators are obtained as shown in Fig. 12.

The optimized GM(0,N) model achieves the lowest RMSE and MAE values across all approaches, which is consistent with the earlier MRE analysis. This confirms that the optimized method proposed in this study provides the best overall performance. For the GM(0,N) and GM(1,N) models, the GM(0,N) model demonstrates better RMSE and MAE values for the fitting samples; however, for the validation samples, the GM(1,N) model outperforms the GM(0,N) model. This suggests that the GM(0,N) model exhibits a certain degree of overfitting, although this issue is completely eliminated after applying the optimization approach developed in this study. The GM(1,1) model shows consistently larger RMSE and MAE values than the three methods mentioned above, while An’s model has the poorest fitting and prediction accuracy, which is also in agreement with the MRE-based analysis presented earlier.

An’s model divides the roundabout into detector-controlled, signal-controlled, and other approaches, and develops separate queue length prediction formulas for these three types. The formulas consider several major factors influencing queue length, including green time, red time, conflicting traffic volume, arriving traffic volume, and other intersection parameters. However, in the comparative analysis, An’s model demonstrated the lowest prediction accuracy. This is mainly because the model multiplies all influencing factors together and applies simple parameter corrections; as a result, the structure of the formula is overly simplified and cannot adapt well to the variability of queue length data at roundabouts.

In contrast, grey models inherently possess advantages in their modeling mechanisms, which enable higher prediction accuracy. Nevertheless, the GM(1,1) model does not incorporate influencing factors, leading to lower accuracy compared with the other grey models. This indicates that considering external influencing factors is also essential for improving prediction performance. Furthermore, the GM(1,N) and GM(0,N) models show similar levels of prediction accuracy. However, based on the overall evaluation metrics, the GM(0,N) model performs better than the GM(1,N) model. The main reason is that GM(1,N) involves more complex modeling procedures and suffers from issues that negatively affect prediction accuracy, such as the nonequivalence between the grey differential equation and the whitening equation, as well as deviations in the computation of the background value sequence. These factors contribute to the unstable prediction behavior of the GM(1,N) model. In contrast, the GM(0,N) model avoids these issues during the modeling process, thereby improving prediction accuracy to some extent. The adaptability of the original queue length sequence to the GM(0,N) model also affects prediction performance. By applying preprocessing techniques to enhance the adaptability of the original sequence, the model’s accuracy can be further improved.

Overall, model adaptability contributes the most to prediction accuracy. Incorporating influencing factors and enhancing the adaptability of the original sequence are also key elements in improving predictive performance. The GM(0,N) model is more suitable for predicting queue lengths at roundabout approaches compared to other models. Furthermore, the optimized GM(0,N) model, which incorporates a transformation of the original sequences, outperforms the standard GM(0,N) model, demonstrating that the proposed optimization approach is both feasible and effective.

From a practical implementation perspective, the proposed optimized GM(0,N) model relies exclusively on variables that are already available in most adaptive traffic control systems. Specifically, the real-time input data required by the model include entry traffic volume, circulating (conflicting) traffic volume, and signal green and red durations. In the field, these variables can be directly obtained from SCATS detector data streams, where vehicle counts are provided by loop detectors or stop-line presence detectors (VS data), and signal phase timing information is available from signal controller logs (SM data). Therefore, no additional sensing infrastructure is required beyond standard SCATS installations.

Although drone-based observations were used in this study to obtain ground-truth queue length measurements for model validation, queue lengths in real-world applications do not need to be measured directly. Instead, the predicted queue length output from the proposed model can be used operationally to support detector placement decisions, such as determining the optimal setback distance of queue detectors on the controlling approach, or to trigger metering signals under adaptive control logic. Alternatively, queue presence can be inferred using conventional point detectors, occupancy ratios, or upstream Bluetooth/Wi-Fi travel time sensors, which are commonly deployed in urban traffic networks.

Therefore, the proposed GM(0,N) model is expected to serve as a valuable platform for detector placement by incorporating real-time variations in green and red signal durations. Moreover, this model can be integrated into adaptive signal control algorithms that dynamically adjust signal phases based on real-time queue length estimations. Unlike conventional fixed-time signal systems, which cannot respond effectively to fluctuations in arrival and conflicting traffic volumes, the proposed model, by integrating signal timing and traffic flow variables, offers the potential to implement real-time feedback mechanisms. This capability allows for dynamic signal timing adjustments to prevent spillback and maintain balanced queue distribution across all approaches.

## Conclusion

This study proposes an optimized GM(0,N) model for real-time prediction of queue lengths at each approach of metered roundabouts. The Grey Model (GM) is capable of reasoning with small and incomplete datasets; To enhance the accuracy of the proposed model, a composite function with two unknown parameters, comprising an exponential function and a trigonometric function, was employed to transform the original sequence. The PSO algorithm was then utilized to determine the optimal values of the unknown parameters, thereby reducing the prediction error. A case study was conducted on the Old Belair Road roundabout, and the results demonstrated that the proposed optimized GM(0,N) model outperformed other models namely An’s model, GM(1,1), GM(1,N), and GM(0,N) across all approaches. Both boxplot analysis of relative errors, MRE, RMSE and MAE analysis confirmed the superior efficiency of the proposed method. These findings suggest that the model can provide accurate queue length predictions during the conversion of conventional roundabouts to metered roundabouts and offer guidance for detector placement strategies. For future research, since grey prediction models are applicable only to small-sample data sequences, the present study collected only one hour of data corresponding to the most congested period of the morning peak. However, the duration of peak-hour traffic is typically much longer than one hour, and factors such as date, weather conditions, surrounding environment, and holidays can all influence queue lengths at roundabouts. Therefore, more influencing factors should be considered, and a larger amount of data should be collected. Furthermore, the analysis is based on data collected from a single intersection during a single morning peak period (07:35–08:35). Therefore, the findings may have limited generalizability across different days, seasons, and other types of metered roundabouts. Under these conditions, the prediction accuracy of grey models can no longer be guaranteed. As a result, future work will explore the application of deep learning methods to address the challenges of predicting queue lengths with large-sample datasets. In addition, comparative analyses with improved Webster model, which predict average queue lengths through signal optimization, and shockwave model, which dynamically predict queue lengths in real-time by analyzing changes in traffic flow as waves, are recommended to further enhance prediction accuracy.

## Data Availability

The datasets used and/or analyzed during the current study available from the corresponding author on reasonable request.
